# Global substance use disorders burden from 1990 to 2021: post-COVID shifts and widening inequalities

**DOI:** 10.7189/jogh.16.04131

**Published:** 2026-04-24

**Authors:** Tianyue Yu, Meiti Wang, Jinxin Zheng, Shiyang Guan, Jianhua Chen

**Affiliations:** 1 Shanghai Mental Health Centre, Shanghai Jiao Tong University School of Medicine, Shanghai Clinical Research Centre for Mental Health, Shanghai Key Laboratory of Psychotic Disorders, Shanghai, China; 2 Shanghai Tenth People's Hospital, Tongji University School of Medicine, Department of Psychiatry, Shanghai, China; 3 Shanghai Jiao Tong University School of Medicine, School of Global Health, Chinese Centre for Tropical Diseases Research, Shanghai, China; 4 Shanghai Jiao Tong University-The University of Edinburgh, One Health Centre, Shanghai, China; 5 Anhui Medical University, School of Public Health, Department of Epidemiology and Biostatistics, Inflammation and Immune-Mediated Diseases Laboratory of Anhui Province, Hefei, China; 6 Xinjiang Medical University, The First Teaching Hospital, Department of Psychiatry, Urumqi, China; 7 Shanghai Institute of Traditional Chinese Medicine for Mental Health, Shanghai, China

## Abstract

**Background:**

Global evidence on substance use disorders (SUDs) remains fragmented, and fewer analyses have benchmarked country-level burden against development gradients. We used Global Burden of Disease 2021 estimates to update long-term trends and inequalities in SUDs, including alcohol use disorder and drug use disorders (DUDs), from 1990 to 2021.

**Methods:**

We analysed Global Burden of Disease 2021 estimates for SUDs, alcohol use disorder, and DUDs across 204 countries and territories. We report age-standardised prevalence rates (ASPR), incidence rates, and disability-adjusted life-years rates (age-standardised disability-adjusted life-years rate) per 100 000 population with 95% uncertainty intervals (UI). Analyses examined temporal trends, age-sex patterns, regional and national variation, socio-demographic index (SDI) – stratified gradients, and deviations of observed burden from SDI-based expected levels.

**Results:**

In 2021, 162.86 million people had SUDs (95% UI = 145.95, 180.85). The global ASPR was 1982.13 per 100 000 (95% UI = 1773.57, 2206.29), declining by 16.9% (95% UI = −18.7, −14.9) since 1990; age-standardised incidence rate was 843.37 (95% UI = 736.21, 956.19), declining by 17.3% (95% UI = −19.7, −15.2); and ASDR was 393.36 (95% UI = 323.56, 469.04), declining by 9.1% (95% UI = −11.9, −5.7). Alcohol use disorder accounted for about 111.12 million cases (95% UI = 96.35, 127.90) (ASPR = 1335.43; 95% UI = 1153.65, 1539.75), and DUDs for about 53.12 million (95% UI = 46.99, 60.95) (ASPR = 663.80; 95% UI = 584.52, 766.14). Geographic heterogeneity was marked: high-income North America had the highest ASDR = 2193.48 (95% UI = 1861.45, 2529.02) and Western Sub-Saharan Africa the lowest ASDR = 136.22 (95% UI = 108.18, 170.26). Drug use disorder burden increased in several high-SDI settings in 2020–2021, while burden peaked in young adults (approximately 25–35 years) and was consistently higher among males.

**Conclusions:**

Despite declining global age standardised rates since 1990, SUDs remain a major source of health loss, with pronounced age–sex differences, substantial cross-country heterogeneity, and widening divergence by development level. Recent increases in drug-related burden in some high-SDI settings highlight the need for strengthened surveillance and context-specific prevention, treatment, and harm-reduction responses.

Substance use disorders (SUDs) are a major public health challenge, contributing substantially to morbidity, premature mortality, and socioeconomic loss. Updated, comparable epidemiological evidence is essential to guide targeted prevention, treatment, and harm-reduction strategies across heterogeneous health-system and policy contexts.

Recent global analyses indicate that SUD burden remains high and unevenly distributed across regions and demographic groups. Drug use disorders (DUDs) have risen or declined only slowly in many settings, with particularly high burdens reported in high Socio-demographic Index (SDI) locations and among males [1,2], whereas alcohol use disorder (AUD) contributes substantial health loss in young adults in several regions, alongside widening cross-regional inequalities over time [1–3]. However, existing Global Burden of Disease (GBD)-based evidence is often reported by single substances, selected regions, or specific populations, and comparatively fewer studies provide a harmonised assessment of AUD, DUDs, and overall SUDs across countries, age groups, sexes, and SDI strata using consistent metrics [2]. Moreover, country-level benchmarking of observed burden against SDI-based expected levels remains limited, leaving uncertainty about which locations most consistently deviate from development-aligned patterns.

The global SUD landscape has also evolved rapidly in the past decade. The emergence of novel psychoactive substances has diversified exposure and risk profiles in multiple settings [4–6]. In addition, the COVID-19 period coincided with disruptions to health services and substance markets that may have reshaped patterns of use and harm, although effects are likely to vary by context [7]. These considerations underscore the need for updated descriptive assessments in the most recent years available.

Therefore, using GBD 2021 estimates from 1990 to 2021, our primary aim was to quantify the global, regional, and national burden of SUDs, AUD, and DUDs [8]. Secondary aims were to characterise age-sex patterns, assess SDI-stratified gradients, and benchmark observed burden against SDI-based expected levels to identify locations and populations that may warrant prioritised prevention and response.

**Adherence to JoGH’s Guidelines for Reporting Analyses of Big Data Repositories Open to Public**

We followed the Journal of Global Health Guidelines for reporting secondary analyses of publicly available large international data repositories (GRABDROP) [9]. Detailed adherence to each GRABDROP item is provided in Table S1 in the **Online Supplementary Document**).

## METHODS

### Data source, study design, and outcomes

This is a secondary analysis of publicly available estimates from the GBD 2021 coordinated by the Institute for Health Metrics and Evaluation [8]. We extracted annual estimates using the GBD Results Tool for SUDs and two major subcategories – AUD and DUDs – for 204 countries and territories from 1990 to 2021 via the GBD Results (Viz Hub) platform. We examined three epidemiological outcomes – incidence, prevalence, and disability-adjusted life-years (DALYs), and reported age-standardised rates (ASRs) per 100 000 population including the age-standardised incidence rate (ASIR), age-standardised prevalence rate (ASPR), and age-standardised DALY rate (ASDR). All estimates are presented with 95% uncertainty intervals (UI). Cause definitions followed the GBD 2021 cause hierarchy and case definitions as implemented by Institute for Health Metrics and Evaluation: SUDs comprise AUDs and DUDs, and DUDs are modelled for major drug classes within the GBD framework using mapped International Classification of Diseases (ICD)/Diagnostic and Statistical Manual of Mental Disorders (DSM) diagnostic definitions [10]. Ethics approval and informed consent were not required because the analyses used de-identified, aggregated estimates from publicly available sources.

### Data handling, age standardisation, and analytical settings

Data management consisted of reproducible extraction and harmonisation of GBD 2021 outputs: filtering to prespecified causes (SUDs, AUD, DUDs), years (1990–2021), locations (204 countries and territories), and age/sex strata; harmonising variable labels and units across tables and figures; and conducting basic consistency checks to confirm availability of estimates and 95% UI across extracted strata. Age-standardised rates were obtained directly from GBD 2021 and are standardised to the GBD world standard population, enabling comparisons across locations and over time [8]. We used GBD 2021 outputs as released by Institute for Health Metrics and Evaluation and did not modify underlying disease models (*e.g.* DisMod-MR 2.1) or default modelling assumptions; analyses were limited to downstream summarisation and visualisation. Uncertainty intervals in GBD are derived from the draw-level estimation framework; we report 95% UIs as provided by GBD 2021 (2.5th–97.5th percentiles across draws). Percentage changes were calculated from point estimates, and where uncertainty bounds for changes were reported, they were derived consistently from the corresponding draw distributions.

### Study period, subgroup analyses, and SDI assessment

We analysed 1990–2021 to match the temporal coverage of GBD 2021 and to ensure comparability of estimates across years, locations, and subgroups within a single GBD iteration [8]. We implemented subgroup analyses by stratifying estimates by GBD region, country/territory, sex, and age group, and summarised patterns across SDI strata. Socio-demographic index is a composite measure of development based on lag-distributed income per capita, average years of schooling, and total fertility rate [11]. To evaluate development gradients, we assessed SDI-rate associations and quantified deviations of observed burden from SDI-based expected levels to identify locations with higher-than-expected burden for their development level. All cross-location and subgroup comparisons are descriptive; we did not perform formal hypothesis testing, and overlapping UIs were not interpreted as evidence for or against statistically meaningful differences. All analyses and figure production were conducted in *R*, version 4.2.2 (R Core Team, Vienna, Austria).

## RESULTS

### Global burden

In 2021, an estimated 162.86 million people had SUDs (95% UI = 145.95, 180.85 million) (**Table 1**; Figure S1 in the **Online Supplementary Document**). Global ASPR = 1982.13 (95% UI = 1773.57, 2206.29), ASIR = 843.37 (95% UI = 736.21, 956.19), and ASDR = 393.36 (95% UI = 323.56, 469.04) per 100 000, corresponding to changes of −16.9%, −17.3%, and −9.1% since 1990 (**Table 1**; Tables S2 and S3 in the **Online Supplementary Document**). Alcohol use disorder comprised 111.12 million cases (95% UI = 96.35–127.90 million), with ASPR = 1335.43, ASIR = 673.98, and ASDR = 202.39 per 100 000, declining by −21.3%, −19.3%, and −23.9%, respectively (Tables S4–6 in the **Online Supplementary Document**). Drug use disorders affected 53.11 million people (95% UI = 46.99, 60.95 million); ASPR and ASIR decreased to 663.80 and 169.39 per 100 000 (changes −6.4% and −8.1%), whereas ASDR increased to 190.97 per 100 000 (+14.7%) (Tables S7–9 in the **Online Supplementary Document**).

**Table 1:** The prevalence counts and age-standardised prevalence rates of substance use disorders, by region, 1990 and 2021

Region	1990		2021		Percentage change in ASRs
	**N (95% UI)**	**ASRs per 100 000 (95% UI)**	**N (95% UI)**	**ASRs per 100 000 (95% UI)**	
Global	122 992 262 (108 382 129, 137 790 871)	2385.67 (2111.67, 2655.64)	162 860 673 (145 950 893, 180853933)	1982.13 (1773.57, 2206.29)	−16.9 (−18.7, −14.9)
Andean Latin America	680 536 (588 758, 775 012)	2077.84 (1817.84, 2338.53)	1 105 501 (963 699, 1 256 340)	1608.00 (1408.60, 1819.84)	−22.6 (−26.2, −18.4)
Australasia	840 506 (764 986, 927732)	3893.36 (3544.31, 4307.41)	1 213 607 (1 097 993, 1 347 402)	3837.44 (3457.67, 4276.76)	−1.4 (−7.4, 3.9)
Caribbean	866 425 (746 789, 997 897)	2548.31 (2207.24, 2895.80)	1 200 874 (1 053 699, 1 355 682)	2399.84 (2105.16, 2716.56)	−5.8 (−9.8, 1.6)
Central Asia	2 325 963 (2 022 869, 2 653 328)	3742.86 (3250.39, 4204.48)	3 082 751 (2 735 368, 3 474 089)	3132.11 (2772.10, 3506.27)	−16.3 (−21.5, −9.5)
Central Europe	4 431 181 (3 967 579, 4 888 041)	3283.42 (2933.19, 3626.10)	4 141 632 (3 739 810, 4 557 115)	3038.29 (2741.78, 3368.37)	−7.5 (−10.2, −4.4)
Central Latin America	3 928 859 (3 429 321, 4 486 705)	2785.28 (2454.00, 3136.54)	6 476 709 (5 742 512, 7 177 674)	2421.63 (2147.95, 2681.26)	−13.1 (−15.6, −10.0)
Central sub-Saharan Africa	574 309 (483 774, 666 684)	1400.55 (1206.21, 1611.29)	1 494 578 (1 261 311, 1 732 543)	1377.07 (1186.13, 1566.99)	−1.7 (−7.6, 5.3)
East Asia	26 840 118 (23 584 660, 30 172 155)	2019.25 (1785.78, 2256.52)	27 678 863 (24 444 044, 31 425 344)	1734.30 (1526.85, 1974.22)	−14.1 (−17.3, −10.8)
Eastern Europe	12 030 502 (10 728 543, 13 432 490)	4818.64 (4287.12, 5375.36)	10 439 295 (9 427 063, 11 608 307)	4287.03 (3857.26, 4737.45)	−11.0 (−14.4, −7.7)
Eastern sub-Saharan Africa	2 594 451 (2 199 873, 2 979 771)	1929.62 (1680.12, 2189.49)	6 185 528 (5 298 175, 7 095 656)	1827.36 (1579.79, 2071.10)	−5.3 (−8.5, −2.0)
High-income Asia Pacific	3 692 144 (3 231 659, 4 224 229)	1960.96 (1717.30, 2247.38)	3 475 196 (3 064 914, 3 870 511)	1823.43 (1585.84, 2095.85)	−7.0 (−13.3, −1.7)
High-income North America	13 786 780 (12 336 004, 15 406 107)	4591.45 (4096.01, 5151.28)	21 007 836 (19 435 365, 22 801 311)	5692.86 (5240.11, 6200.79)	24.0 (17.3, 32.4)
North Africa and Middle East	2 382 982 (2 066 826, 2 712 149)	787.89 (685.44, 884.11)	5 189 728 (4 540 378, 5 795 137)	802.04 (706.09, 892.51)	1.8 (−0.5, 4.3)
Oceania	89 569 (73 137, 108 261)	1484.02 (1247.38, 1746.78)	204 798 (171 930, 243 218)	1520.19 (1290.80, 1779.11)	2.4 (−3.4, 8.9)
South Asia	19 349 935 (16 511 579, 22 366 099)	2101.59 (1802.84, 2422.69)	30 606 515 (26 858 861, 34 727 519)	1628.57 (1433.64, 1842.20)	−22.5 (−26.2, −18.7)
Southeast Asia	5 873 463 (5 032 825, 6 809 811)	1317.88 (1146.56, 1505.40)	9 436 641 (8 221 982, 10 697 547)	1265.05 (1104.30, 1436.39)	−4.0 (−6.6, −1.2)
Southern Latin America	1 523 127 (1 315 470, 1 763 769)	3130.61 (2705.85, 3623.40)	2 102 614 (1 834 020, 2 392 307)	2901.97 (2531.27, 3313.45)	−7.3 (−13.0, −0.5)
Southern sub-Saharan Africa	1 252 318 (1 089 453, 1 420 482)	2854.55 (2502.00, 3227.33)	2 082 767 (1 815 334, 2 364 271)	2575.43 (2263.77, 2901.73)	−9.8 (−12.8, −6.9)
Tropical Latin America	5 395 433 (4 639 144, 6 137 937)	3733.94 (3227.43, 4227.45)	8 549 945 (7 602 155, 9 639 741)	3398.54 (3024.48, 3850.07)	−9.0 (−14.1, −3.3)
Western Europe	13 401 863 (12 070 760, 14 863 718)	3277.32 (2940.62, 3661.61)	14 307 681 (13 015 459, 15 606 529)	3270.09 (2959.60, 3624.71)	−0.2 (−3.1, 2.9)
Western sub-Saharan Africa	1 131 796 (983 976, 1 294 866)	763.70 (669.06, 860.69)	2 877 613 (2 499 207, 3 283 208)	746.93 (655.91, 839.74)	−2.2 (−4.0, −0.2)

ASR – age-standardised rate, UI – uncertainty interval

Burden trends diverged by SDI across all three metrics (Tables S10–12 in the **Online Supplementary Document**). High SDI regions were the only stratum with increases in SUD ASPR (+10.2%), ASIR (+46.8%), and ASDR (+93.9%), while other strata generally declined (Table S10 in the **Online Supplementary Document**). For AUDs, ASPR/ASIR/ASDR decreased across all SDI strata, with the largest reductions typically in high-middle SDI settings (Table S11 in the **Online Supplementary Document**). For DUDs, the development gradient was most pronounced: high SDI regions showed marked increases in ASPR (+47.9%), ASIR (+62.6%), and ASDR (+238.5%), whereas middle and high-middle SDI strata declined and low SDI strata were broadly stable (Table S12 in the **Online Supplementary Document**).

### Region burden

In 2021, substantial regional heterogeneity in SUDs burden persisted (Tables S1–3 in the **Online Supplementary Document**). High-income North America had the highest ASPR = 5692.86 per 100 000, 95% UI = 5240.11, 6200.79), with similarly high ASIR and ASDR, followed by Eastern Europe (ASPR = 4287.03, 95% UI = 3857.26, 4737.45) and Australasia (ASPR = 3837.44, 95% UI = 3457.67, 4276.76). The lowest ASPR was observed in North Africa and the Middle East (ASPR = 802.04, 95% UI = 706.09, 892.51) and Western Sub-Saharan Africa (ASPR = 746.93, 95% UI = 655.91–839.74). Since 1990, increases in SUDs ASPR were largely confined to high-income North America (+24.0%, 95% UI = 17.3, 32.4) and Oceania (+2.4%, 95% UI = −3.4, 8.9), whereas most other regions declined (Table S1 in the **Online Supplementary Document**).

For AUD, ASPR decreased in most regions but increased in Australasia (+20.7%, 95% UI = 8.7, 33.8). The highest AUD ASPR occurred in Eastern Europe (ASPR = 3292.73, 95% UI = 2901.33, 3724.07) and Central Asia (ASPR = 2581.06, 95% UI = 2252.39, 2969.55) (Table S4 in the **Online Supplementary Document**). Drug use disorders showed the strongest divergence across ASPR, ASIR, and ASDR. High-income North America recorded marked increases in ASPR to 3668.00 (95% UI = 3323.49, 4067.36) and an exceptional rise in ASDR (+421.5%, 95% UI = 372.2, 483.4), the highest globally, whereas East Asia showed substantial declines in both ASPR (−27.2%, 95% UI = −31.5, −22.0) and ASDR (−56.3%, 95% UI = −61.8, −51.6) (Table S3 in the **Online Supplementary Document**). Overall, regional declines in SUD burden co-occurred with steeply rising drug-related harm in selected high-SDI regions.

### National burden

**Figure 1:**
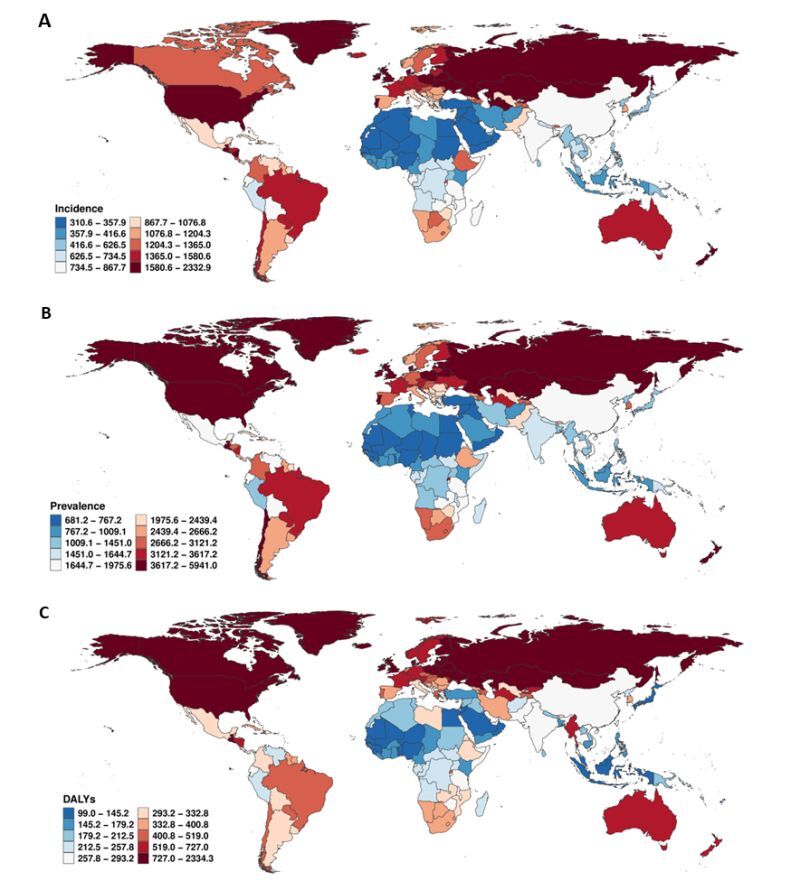
Global age standardised incidence, prevalence, and DALY rates of substance use disorders, 2021. **Panel A**. Age standardised incidence. **Panel B**. Age standardised prevalence. **Panel C**. DALY rates. DALY – disability adjusted life year.

Marked cross-national heterogeneity was observed for SUDs and both subcomponents (**Figure 1**, Panels A–C; Figures S2 and S3 in the **Online Supplementary Document**). For SUDs, ASPR ranged from 5882.23 (95% UI = 5412.35, 6418.30) per 100 000 in the USA and 5286.32 (95% UI = 4550.70–6033.04) in Mongolia to <730 per 100 000 in several Middle Eastern countries, while ASDR varied from 2311.18 (95% UI = 1958.69, 2672.95) in the USA to <100 per 100 000 in Indonesia. For AUD, the highest ASPR occurred in Mongolia (ASPR = 4716.94, 95% UI = 3986.35, 5453.81) and Guatemala (ASPR = 4049.97, 95% UI = 3444.01, 4623.66), with particularly low burden in North Africa and the Middle East (*e.g.* Iran ASDR = 39.13, 95% UI = 28.84, 52.26). Drug use disorders showed the most pronounced inequality: ASPR peaked in the USA (ASPR = 3821.43, 95% UI = 3450.13, 4257.62) and remained high in Canada and the UK; ASIR exceeded 500 per 100 000 in the USA and Australia, whereas estimates were < 230 per 100 000 across much of sub-Saharan Africa and South Asia. Drug use disorders ASDR exceeded 1900 per 100 000 in the USA but was <40 per 100 000 in several West African countries. Given cross-country differences in surveillance and attribution, national contrasts – particularly very low DUDs estimates – should be interpreted as descriptive patterns rather than definitive rankings.

### Age- and sex-specific patterns of SUD burden

**Figure 2:**
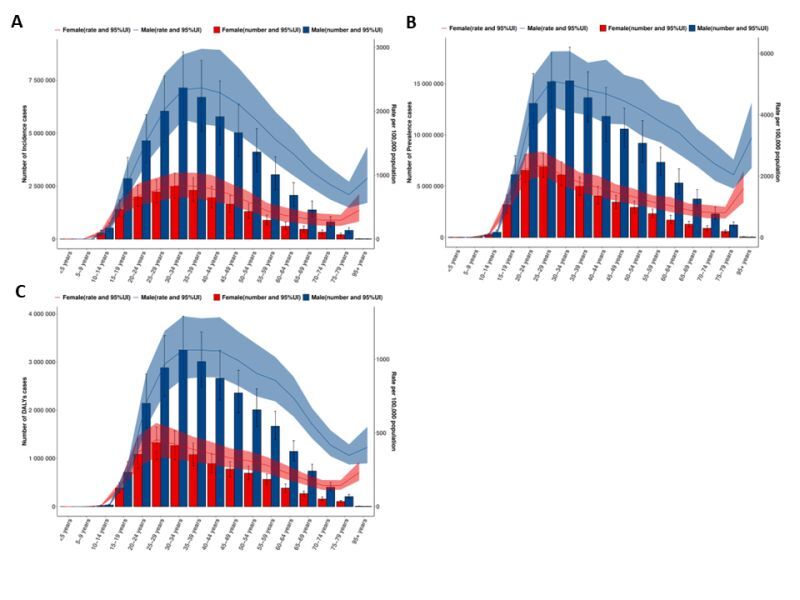
Age sex patterns of substance use disorder burden, 2021. **Panel A**. Incidence. **Panel B**. Prevalence. **Panel C**. DALYs. DALY – disability adjusted life year.

Pronounced age-sex gradients were observed for SUDs, AUD, and DUDs (**Figure 2****,** Panels A–C; Figures S4 and S5 in the **Online Supplementary Document**). For SUDs, males had higher rates than females across most ages, with the largest gaps in young-to-middle adulthood. ASPR peaked at 25–29 years (males ASPR = 5107.77; 95% UI = 4261.63, 6068.98 *vs.* females ASPR = 2368.65; 95% UI = 2017.59, 2805.50), ASIR peaked at 30–34 years (males ASIR = 2336.07; 95% UI = 1865.87, 2889.60 *vs.* females ASIR = 833.16; 95% UI = 647.59, 1038.79), and ASDR was highest at 30–34 years in males (ASDR = 1063.17; 95% UI = 867.11, 1292.75) and 25–29 years in females (ASDR = 455.36; 95% UI = 337.07, 567.43).

For AUD, patterns were broadly similar – ASPR peaked at 40–44 years (males ASPR = 3858.79; 95% UI = 2892.46, 4975.50 *vs.* females ASPR = 998.22; 95% UI = 711.86, 1383.06), whereas ASDR peaked later in midlife (males 45–49 years, ASDR = 688.89; 95% UI = 544.20, 877.48; females 40–44 years, ASDR = 137.58; 95% UI = 99.34, 192.09). By contrast, DUDs peaked earlier: ASPR was highest at 20–24 years (males ASPR = 2247.83; 95% UI = 1840.37, 2817.94 *vs.* females ASPR = 1449.14; 95% UI = 1192.41, 1832.22), ASIR peaked at 15–19 years in males (ASIR = 393.8; 95% UI = 306.3, 508.5) and 20–24 years in females (ASIR = 285.6; 95% UI = 226.2, 356.0), and ASDR peaked at 25–29 years in both sexes (males ASDR = 573.20; 95% UI = 464.30, 679.97 *vs*. females ASDR = 345.03; 95% UI = 259.70, 434.73). Estimates at the oldest ages – including the late-life rise in SUDs/AUD rates and the apparent female-male crossover for DUDs after age 70 – should be interpreted cautiously given sparse input data and wider uncertainty.

### Trends and correlations of disease burden by SDI

**Figure 3:**
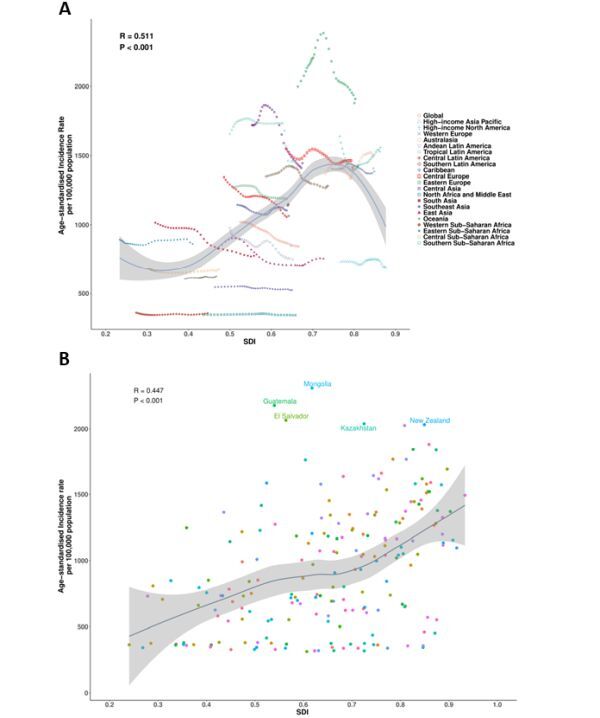
Association between SDI and age standardised incidence of substance use disorders, 2021. **Panel A**. Twenty-one Global Burden of Disease regions. Each point represents one region; colours correspond to super regions. **Panel B**. Two hundred and four countries and territories. Selected countries with notably higher than expected incidence are labelled. SDI – sociodemographic index.

Sociodemographic index gradients differed by substance (**Figure 3**, Panels A–C; Figures S6–13 in the **Online Supplementary Document**). Regionally, SUDs and AUD showed a nonlinear SDI pattern – lowest at SDI<0.4, peaking at 0.4–0.8, and declining at SDI > 0.8, with modest Pearson correlations for ASPR/ASIR/ASDR (SUDs: R = 0.609 / 0.511 / 0.478; AUDs: R = 0.415 / 0.400 / 0.283; all *P* < 0.001). By contrast, DUDs exhibited monotonic positive associations with SDI (R = 0.677 / 0.731 / 0.526; *P* < 0.001). Australasia and high-income North America consistently lay above SDI-based expectations for SUDs and DUDs, whereas high-income Asia Pacific tended to fall below, and Mongolia, Guatemala, and El Salvador were prominent outliers for AUDs.

National patterns were similar. Correlations for SUDs remained moderate (R = 0.509 / 0.447 / 0.469; *P* < 0.001) and were weaker for AUDs (R = 0.368 / 0.368 / 0.286; *P* < 0.001), while DUDs showed the strongest SDI associations (R = 0.765 / 0.835 / 0.707; *P* < 0.001). Countries with higher-than-expected burden clustered within a small set of locations, including Mongolia and several high-income settings; the USA was the most pronounced outlier for DUD ASPR and ASDR, and also exceeded expectations for ASIR alongside countries such as Australia, Canada, Estonia, and New Zealand. Conversely, high-income Asia Pacific countries (*e.g.* Japan, Singapore, South Korea) generally fell below SDI-based expectations.

## DISCUSSION

Using GBD 2021 estimates, we quantified the global, regional and national burden of SUDs, and provided substance-specific analyses for AUDs and DUDs from 1990 to 2021 by age, sex and SDI. Key findings indicate that SUDs affected a substantial global population in 2021 and that age-standardised prevalence and incidence have declined since 1990, whereas the reduction in overall health loss was more modest; concentration of burden in young adults (incidence and DALYs peaking in the mid-20s to mid-30s); and substantially higher ASIR, ASPR and ASDR among males across most age groups. The results also highlight two linked patterns: apparent deviations from prior trajectories around 2020, notably increases in DUDs burden and mortality in several high SDI settings, and widening inequalities in substance-attributable health loss across SDI strata, reflected in divergent ASPR and ASDR trends and multiple country-level outliers (*e.g.* high-income North America, parts of Eastern Europe). Compared with earlier assessments based on data up to 2010, current GBD estimates show measurable declines in several burden metrics, a pattern that is consistent with improvements in prevention and treatment in some settings, but requires formal evaluation for attribution [12].

### Global burden and temporal changes

Improvements in public health programming and treatment access have been reported to reduce substance related harm in some settings [13,14]. Several interventions have been evaluated in specific settings (*e.g.* naloxone distribution, harm-reduction services, and telemedicine-enabled models of care). However, our analyses did not incorporate policy coverage, implementation intensity, or service-access indicators, and therefore these interventions are discussed here only as contextual background rather than explanations for the observed temporal changes. Expanded naloxone availability [15], implementation of needle exchange and supervised consumption services [16], and wider use of telemedicine during the COVID-19 pandemic have been associated with reductions in overdose mortality and improved continuity of care [17]. Accordingly, interpretation of the observed trends should remain descriptive, and future work should explicitly link burden estimates to policy timelines and service-coverage metrics to enable rigorous impact evaluation and attribution.

### Regional and national heterogeneity

Regional disparities in SUDs burden reflect substantial heterogeneity in exposure, health system capacity, and policy environments. In 2021, high-income North America had the highest ASDR, whereas Western Sub-Saharan Africa had the lowest, highlighting a >16-fold difference across regions. These contrasts cannot be causally explained by our ecological, modelled estimates, but they can help prioritise settings for strengthened surveillance and evaluation. Policy and market factors may contribute to these contrasts. For example, in some jurisdictions, tighter opioid prescribing has been associated with shifts toward illicit synthetic opioids and changing overdose profiles [18]. Similar patterns have also been reported in other high-income settings, where changes in drug supply and service access have shaped recent mortality trends [19]. In addition, deindustrialisation and social disadvantage have been implicated in localised increases in DUDs mortality in parts of the USA [20]. The persistent high burden of alcohol-attributable harm in parts of Eastern Europe has been related to entrenched cultural drinking patterns and socioeconomic transition [21–23]. These contextual explanations should be interpreted as hypotheses informed by external evidence rather than direct causal inferences from our GBD-based analyses; causal attribution would require country-level evaluations integrating policy timelines, treatment coverage, and supply-side indicators. Consistent with the descriptive patterns observed in our results, potential priorities include strengthening real-time monitoring and expanding evidence-based responses tailored to local epidemiology; however, decisions about scale-up should be supported by setting-specific implementation data and impact evaluation.

### Age-sex patterns and late-life uncertainty

Our results confirm marked age- and sex-related patterns: young adults (approximately 25–35 years) bore the largest share of SUDs prevalence and DALYs, and males had substantially higher ASIR, ASPR, and ASDR across most ages [24,25]. Social and structural factors (including stigma and gendered differences in care-seeking), together with shared genetic liability and neurodevelopmental vulnerabilities, might contribute to these disparities [26–30]. These factors are not measured in our analyses and are therefore presented as hypotheses derived from prior work, rather than as explanations supported by our data.

Two age-specific findings warrant particular caution. First, the higher estimated SUDs/AUD prevalence and incidence at very old ages (after 75 years) could reflect late-onset exposure or cohort effects, but may also arise from measurement and modelling limitations in older populations [31,32], including multimorbidity-related misclassification, survivorship bias, and sparse input data yielding wider uncertainty. Second, the estimated crossover in DUDs burden among women aged over 70 years differs from common epidemiological descriptions and should be interpreted as hypothesis-generating rather than definitive. Potential explanations include differential diagnosis or recording by sex in late life, selective survival, and higher uncertainty in extreme age strata. Given the ecological and modelled nature of GBD outputs and the absence of additional validation within this study, these late-life patterns require confirmation using individual-level longitudinal data with consistent case definitions and recoding checks where feasible.

### SDI gradients and observed-to-expected deviations

Our SDI-stratified findings suggest that development shapes the composition of SUDs burden rather than shifting all substances in the same direction. Alcohol use disorder-related burden appears to follow an inverted-U pattern across SDI, whereas drug-related harm increases with development level, implying that different policy and health-system levers may be required across settings. Importantly, recent increases in drug-related burden in several high-SDI locations highlight a divergence that is not captured by global averages, and underscore the need to interpret ‘progress’ cautiously when it coexists with worsening outcomes in specific settings or drug classes [33].

Several hypotheses can account for these patterns. From a true epidemiology perspective, high SDI contexts may experience greater availability of synthetic and prescription drugs, higher disposable income enabling access, and social determinants (urbanisation, social isolation, economic pressures) that raise risk exposures and comorbid mental health conditions [34–36]. These factors plausibly contribute to higher DUDs burden in some wealthy settings. Conversely, in low SDI settings the observed lower burden may reflect both lower exposure in some contexts and substantial under ascertainment due to limited surveillance and health care access; hence, comparisons must consider differential data completeness.

Observed temporal inflections around 2020, particularly the rise in DUDs burden and mortality in several high SDI settings, are temporally consistent with documented pandemic-related service disruptions, supply side shifts and increases in overdose deaths [37–39]. However, we did not perform causal interrupted time series analyses and cannot attribute these changes to the pandemic on the basis of our data alone [40]. Possible contributors proposed in prior studies include reduced access to treatment (clinic closures, service reallocation), interruptions to harm reduction services, shifts in illicit drug supply toward more potent synthetic opioids, and broader psychosocial stressors during lockdowns; however, these mechanisms were not tested in our study and should be treated as hypotheses requiring quasi-experimental evaluation [41]. Accordingly, post-COVID policy responses should be framed as priorities for monitoring and evaluation – protecting continuity of care, strengthening supply-side intelligence, and linking clinical/prescription/toxicology data for surveillance – rather than as causal interpretations of the observed inflections.

Our findings imply distinct policy priorities across settings and underscore the need for post-COVID resilience in addiction systems. In high SDI contexts, DUDs burden and mortality increased in recent years, policy emphasis should be on ensuring continuity and surge capacity of evidence-based treatment, expanding low threshold harm reduction and strengthening real-time surveillance that links clinical, prescription and toxicology data to supply-side intelligence. In mid-SDI settings rising AUD or DUDs indicators call for reinforced prevention, calibrated fiscal and marketing measures, and improved monitoring to guide locally appropriate interventions. In low-SDI contexts, priorities include investment in basic surveillance, integration of addiction care into primary health services, and international technical support to improve case detection and management [42,24]. Across all strata, pandemic preparedness must explicitly protect addiction services and build rapid response surveillance and evaluation capacity; targeted experimental evaluations should accompany policy roll outs to assess impact and guide scale up.

### Tailoring interventions for vulnerable populations

Beyond SDI-stratified priorities, interventions should be adapted for vulnerable populations. For children and adolescents, emphasis should be placed on early prevention and age-appropriate identification (school- and family-based programmes, restrictions on youth-targeted alcohol marketing and availability, and clear referral pathways to youth mental health/addiction services) [43]. For individuals with comorbidities, policies should prioritise integrated care models that link SUD treatment with primary care and mental health services to improve continuity and reduce relapse and overdose risk [44]. For socioeconomically disadvantaged communities, implementation should lower structural barriers through low-threshold access, community outreach, wider naloxone distribution, and linkage to housing and social support [33]. These implementation considerations are informed by external evidence; in the context of our descriptive GBD analysis, they should be interpreted as options for prioritisation and evaluation rather than direct implications derived from our results.

### Strengths and limitations

This study provides a version-locked, substance-specific and country-level assessment of SUDs using GBD 2021 estimates for 204 countries and territories from 1990 to 2021. By jointly reporting SUDs, AUDs, and DUDs across age, sex, and SDI strata, and presenting estimates with 95% UIs, it offers a coherent description of long-term trends and contemporary geographic heterogeneity. The integration of development gradients and observed-to-expected comparisons helps identify settings where burden is disproportionate relative to development level and may inform priority areas for prevention, treatment, and harm reduction.

Several limitations warrant consideration. Substance use disorders may be under-ascertained because stigma and criminalisation can reduce disclosure, introducing social desirability bias, and variation in diagnostic practices can lead to misclassification, particularly across age and sex strata. Our analyses involve many subgroup comparisons, so some extreme estimates may reflect multiple testing and should be interpreted alongside uncertainty intervals and consistency across metrics. The analyses used published GBD 2021 aggregated outputs; therefore, we did not perform additional missing-data imputation or record-level corrections, and causal attribution of temporal changes (including around 2020) is not possible without dedicated designs incorporating policy and supply-side information.

## CONCLUSIONS

This global analysis of GBD 2021 estimates (1990–2021) shows that, despite overall declines in ASRs, substance use disorders remain a major source of health loss, concentrated in young adults and consistently higher among males. Importantly, global averages mask diverging trajectories by development level: several high-SDI settings show recent increases in drug-related burden and mortality, alongside rapid growth in stimulant-related disorders (including amphetamines and cocaine) in selected regions. These findings support targeted responses – strengthening real-time surveillance and scaling evidence-based treatment and harm reduction where DUD burden is rising, while reinforcing prevention and integrating addiction care into primary health systems in middle- and low-SDI settings, supported by routine monitoring and rigorous impact evaluation.

## Additional material


Online Supplementary Document
