# Supplementary material for: Global substance use disorders burden from 1990 to 2021: post-COVID shifts and widening inequalities
Source: J Glob Health. 2026 Apr 24;16:04131. doi: 10.7189/jogh.16.04131 (PMC13107084; doi:10.7189/jogh.16.04131)
Supplement: Online Supplementary Document [file jogh-16-04131-s001.pdf]

Supplement to: Yu T, Wang M, Zheng J, Guan S, Chen J. Global substance use disorders burden from 1990 to 2021: post-COVID shifts and widening inequalities. J Glob Health. 2026;16:04131.

Table legend

Table S1. Adherence to JoGH's GRABDROP guidelines items.
Table S2. The incidence counts and age-standardised incidence rates of substance use disorders, by region, 1990 and 2021.
Table S3. The DALYs counts and age-standardised DALY rates of substance use disorders, by region, 1990 and 2021.
Table S4. The prevalence counts and age-standardised prevalence rates of alcohol use disorders, by region, 1990 and 2021.
Table S5. The incidence counts and age-standardised incidence rates of alcohol use disorders, by region, 1990 and 2021.
Table S6. The DALYs counts and age-standardised DALY rates of alcohol use disorders, by region, 1990 and 2021.
Table S7. The prevalence counts and age-standardised prevalence rates of drug use disorders, by region, 1990 and 2021.
Table S8. The incidence counts and age-standardised incidence rates of drug use disorders, by region, 1990 and 2021.
Table S9. The DALYs counts and age-standardised DALY rates of drug use disorders, by region, 1990 and 2021.
Table S10. The prevalence, incidence, and DALY counts and age-standardised rates of substance use disorders, by SDI region, 1990 and 2021.
Table S11. The prevalence, incidence, and DALY counts and age-standardised rates of alcohol use disorders, by SDI region, 1990 and 2021.
Table S12. The prevalence, incidence, and DALY counts and age-standardised rates of drug use disorders, by SDI region, 1990 and 2021.

Figure legend

Figure S1. Age-standardised (A) incidence, (B) prevalence, and (C) DALY rates of substance use disorders by Socio-demographic Index, 1990–2021. Abbreviations: SDI=Socio-demographic Index.
Figure S2. Global age-standardised (A) incidence, (B) prevalence, and (C) DALY rates of alcohol use disorders, 2021. Abbreviations: DALY=disability-adjusted life year.
Figure S3. Global age-standardised (A) incidence, (B) prevalence, and (C) DALY rates of drug use disorders, 2021. Abbreviations: DALY=disability-adjusted life year.
Figure S4. Age-sex patterns of alcohol use disorder burden, 2021. (A) Incidence, (B) Prevalence, (C) DALYs. Abbreviations: DALY=disability-adjusted life year.
Figure S5. Age-sex patterns of drug use disorder burden, 2021. (A) Incidence, (B) Prevalence, (C) DALYs. Abbreviations: DALY=disability-adjusted life year.
Figure S6. Association between Socio-demographic Index and age-standardised prevalence of substance use disorders, 2021. (A) 21 Global Burden of Disease regions. (B) 204 countries and territories. Abbreviations: SDI=Socio-demographic Index.
Figure S7. Association between Socio-demographic Index and age-standardised DALY rates of substance use disorders, 2021. (A) 21 Global Burden of Disease regions. (B) 204 countries and territories. Abbreviations: SDI=Socio-demographic Index; DALY=disability-adjusted life year.
Figure S8. Association between Socio-demographic Index and age-standardised incidence of alcohol use disorders, 2021. (A) 21 Global Burden of Disease regions. (B) 204 countries and territories. Abbreviations: SDI=Socio-demographic Index.

Figure S9. Association between Socio-demographic Index and age-standardised prevalence of alcohol use disorders, 2021. (A) 21 Global Burden of Disease regions. (B) 204 countries and territories. Abbreviations: SDI=Socio-demographic Index.

Figure S10. Association between Socio-demographic Index and age-standardised DALY rates of alcohol use disorders, 2021. (A) 21 Global Burden of Disease regions. (B) 204 countries and territories. Abbreviations: SDI=Socio-demographic Index; DALY=disability-adjusted life year.

Figure S11. Association between Socio-demographic Index and age-standardised incidence of drug use disorders, 2021. (A) 21 Global Burden of Disease regions. (B) 204 countries and territories. Abbreviations: SDI=Socio-demographic Index.

Figure S12. Association between Socio-demographic Index and age-standardised prevalence of drug use disorders, 2021. (A) 21 Global Burden of Disease regions. (B) 204 countries and territories. Abbreviations: SDI=Socio-demographic Index.

Figure S13. Association between Socio-demographic Index and age-standardised DALY rates of drug use disorders, 2021. (A) 21 Global Burden of Disease regions. (B) 204 countries and territories. Abbreviations: SDI=Socio-demographic Index; DALY=disability-adjusted life year.

Table S1. Adherence to JoGH's GRABDROP guidelines items.

**JoGH guideline items**
1. Please list all papers published by each co-author in previous 3 years that were based on secondary analysis of a big data repository
Cheng Y, Fang Y, **Zheng J**, **Guan S**, **Wang M**, Hong W. The burden of depression, anxiety and schizophrenia among the older population in ageing and aged countries: an analysis of the Global Burden of Disease Study 2019. Gen Psychiatr. 2024 Jan 19;37(1):e101078. doi: 10.1136/gpsych-2023-101078
Xu JJ, Ding LY, Sun CC, Qiao Y, **Wang MT, Zheng JX**, Wang G. The burden of mental disorders, substance use disorders, and self-harm among youths globally: findings from the 2021 Global Burden of Disease study. Transl Psychiatry. 2025 Sep 29;15(1):346. doi: 10.1038/s41398-025-03533-x.
**Guan SY, Zheng JX**, Sam NB, Xu S, Shuai Z, Pan F. Global burden and risk factors of musculoskeletal disorders among adolescents and young adults in 204 countries and territories, 1990-2019. Autoimmun Rev. 2023 Aug;22(8):103361. doi: 10.1016/j.autrev.2023.103361
**Wang M**, Jin G, Cheng Y, **Guan SY, Zheng J**, Zhang SX. Genetically predicted circulating levels of cytokines and the risk of depression: a bidirectional Mendelian-randomization study. Front Genet. 2023 Aug 4;14:1242614. doi: 10.3389/fgene.2023.1242614
Zhang SX, Lu ZH, **Wang MT**, Shen YP, Duan L, **Guan SY**, Chen MX, Lu Y, Yang M, Wang L, Yang GB, Lv WW, Wang JC, **Zheng JX**. Assessing the association between the circulating levels of inflammatory cytokines and the risk of tuberculosis: A bidirectional two-sample mendelian randomization study. Infect Genet Evol. 2023 Dec;116:105524. doi: 10.1016/j.meegid.2023.105524
Zhang SX, Wang JC, Yang J, Lv S, Duan L, Lu Y, Tian LG, Chen MX, Liu Q, Wei FN, Feng XY, Yang GB, Li YJ, Wang Y, Hu XJ, Yang M, Lu ZH, Zhang SY, Li SZ, **Zheng JX**. Epidemiological features and temporal trends of the co-infection between HIV and tuberculosis, 1990-2021: findings from the Global Burden of Disease Study 2021. Infect Dis Poverty. 2024 Aug 16;13(1):59. doi: 10.1186/s40249-024-01230-3
**Guan SY, Zheng JX**, Feng XY, Zhang SX, Xu SZ, Wang P, Pan HF. Global burden due to modifiable risk factors for autoimmune diseases, 1990-2021: Temporal trends and socio-demographic inequalities. Autoimmun Rev. 2024 Dec;23(12):103674. doi: 10.1016/j.autrev.2024.103674
Zhu YS, Sun ZS, **Zheng JX**, Zhang SX, Yin JX, Zhao HQ, Shen HM, Baneth G, Chen JH, Kassegne K. Prevalence and attributable health burdens of vector-borne parasitic infectious diseases of poverty, 1990-2021: findings from the Global Burden of Disease Study 2021. Infect Dis Poverty. 2024 Dec 11;13(1):96. doi: 10.1186/s40249-024-01260-x
**Zheng J**, Zhang D, Zhang S, Chen M, Guo Z, Guan S, Liu Y, Bergquist R, Li S, Zhou X, Feng X. Global burden of malaria and neglected tropical diseases in children and adolescents, 1990-2019: a population-based, cross-sectional study. J R Soc Med. 2025 Mar;118(3):82-96. doi: 10.1177/01410768251321572
Zhu H, Zhao M, **Zheng J**. Global, regional, and national burden of retinoblastoma in children aged under 10 years from 1990 to 2021 and projections for future disease burden. Sci Rep. 2025 Mar 3;15(1):7488. doi: 10.1038/s41598-025-91289-1
**Guan SY, Zheng JX**, Zhang SX, Xu S, Shuai Z, Cai HY, Pan F. Global Burden of Musculoskeletal Disorders in Adults Aged 50 and Over, 1990-2021: Risk Factors and Sociodemographic Inequalities. J Cachexia Sarcopenia Muscle. 2025 Aug;16(4):e70008. doi: 10.1002/jcsm.70008
Liu T, Chen Z, Ge J, Mei H, Shi L, **Zheng J**. The evolving burden of childhood meningitis in low- and middle-income countries, 1990-2021: a decomposition and frontier analysis. Eur J Pediatr. 2025 Oct 12;184(11):679. doi: 10.1007/s00431-025-06516-8
**Zheng J**, Tong H, Chen M, Duan L, Song P, Sun J, Zhou X, Feng X. Global burden of dengue

from 1990 to 2021: a systematic analysis from the Global Burden of Disease study 2021. Infect Dis Poverty. 2025 Oct 16;14(1):105. doi: 10.1186/s40249-025-01365-x

Zheng JX, Liu Y, **Guan SY**, Guo ZY, Duan L, Lv S, Zhang SX, Li SZ, Zhou XN, Feng XY. Global, regional, and national burden of neglected tropical diseases and malaria in the general population, 1990-2021: Systematic analysis of the global burden of disease study 2021. J Adv Res. 2026 Jan;79:769-781. doi: 10.1016/j.jare.2025.04.004

Ba H, Zhang D, **Guan S, Zheng J.** Global burden of myocarditis and cardiomyopathy in children and prediction for 2035 based on the global burden of disease study 2019. Front Cardiovasc Med. 2023 May 2;10:1173015. doi: 10.3389/fcvm.2023.1173015

Ma Z, Li B, Qian Y, Mu S, Wang Y, Cui J, Qiu L, **Zheng J**, Li Y, Wen F, Lu Z. Global, regional, and national temporal trend in burden of chronic respiratory diseases from 1990 to 2021: Findings from the Global Burden of Disease Study 2021. Chin Med J (Engl). 2025 Aug 29;138(24):3443–56. doi: 10.1097/CM9.0000000000003670

Liu Y, **Zheng J**, Zhang Y, Pan T, Wang Y, Liu Q, Wang Z, He Q, Yang Y, Zhang H, Kan CS, Liu R, Song L. Aging population of tracheal, bronchus, and lung cancer: global, regional, and national burden-insights from the Global Burden of Disease Study 2021. J Thorac Dis. 2025 Aug 31;17(8):5547-5560. doi: 10.21037/jtd-2025-243

Chen XF, Li Q, Bergquist R, **Zheng JX**, Guo SY, Lan QF, He ZZ, Zhang LJ, Cao CL, Xu J, Zhou XN. Estimation and prediction on the economic burden of schistosomiasis in 25 endemic countries. Infect Dis Poverty. 2025 Jun 16;14(1):49. doi: 10.1186/s40249-025-01330-8

Zhu H, Huang J, **Zheng J**, Zhou C, Zhu T, Zhang M, Zhao L, Wu X, Xue J, Zhou XN, Li S, Qian M. Spatial distribution patterns and risk factors of hookworm disease in China: A study based on successive national surveillance. PLoS Negl Trop Dis. 2025 Sep 30;19(9):e0013526. doi: 10.1371/journal.pntd.0013526

Zhang S, Yang G, Lv S, Duan L, Chen M, Liu Q, Tian L, Li S, **Zheng J**. Global, regional, and national temporal trend and patterns of change in the burden of leishmaniasis from 1990 to 2021: an analysis of the Global Burden of Disease Study 2021. Sci One Health. 2025 Sep 6;4:100123. doi: 10.1016/j.soh.2025.100123

Yu C, **Chen J**. Global Burden of substance use disorders among adolescents during 1990-2021 and a forecast for 2022-2030: an analysis for the Global Burden of Disease 2021. BMC Public Health. 2025 Mar 14;25(1):1012. doi: 10.1186/s12889-025-22107-6.

Sun G, Xia D, Xue B, Jian X, Peng L, Wang B, Wu C, Gao C, He L, Xu Y, Zhao X, Zhang Q, Cao H, Wen Y, Shi Y, Potash JB, **Chen J**, Li Z. Reassessing the relationship between major depressive disorder and blood lipids: a comprehensive Mendelian randomisation study. Gen Psychiatr. 2025 Jun 26;38(3):e101900. doi: 10.1136/gpsych-2024-101900

2. Please explain the key elements of your study design and the use of the available datasets that make your study an original scientific contribution

This study is a secondary analysis of GBD 2021 draw-based estimates for substance use disorders (SUDs) and their two major subcategories—alcohol use disorders (AUDs) and drug use disorders (DUDs)—covering 204 countries and territories from 1990 to 2021. The design was prespecified to (i) quantify long-term changes in age-standardised incidence, prevalence, and DALY rates (ASIR, ASPR, ASDR) for SUDs, AUDs, and DUDs; (ii) characterise age–sex patterns to identify populations bearing the greatest burden; and (iii) interrogate development gradients using the Socio-demographic Index (SDI) through two complementary approaches: SDI-stratified trend comparisons and an observed-versus-expected assessment that identifies locations deviating from SDI-aligned burden trajectories.

The original scientific contribution lies in synthesising these components within a single, harmonised framework and explicitly prioritising interpretation of diverging trajectories that are masked by global averages—most notably the sharp rise in DUD burden in selected high-SDI settings and the marked post-2020 inflections in several regions—while simultaneously contextualising unusually low estimates in data-sparse settings as potentially influenced by

under-ascertainment. In addition, by presenting substance-specific patterns for major drug classes alongside aggregate SUD metrics, the study provides a policy-relevant signal of which components are driving recent divergence. Collectively, these elements move beyond routine descriptive reporting by linking long-term trends, development gradients, and outlier identification to define priority settings and populations for targeted prevention, treatment, and harm-reduction responses.
3. Please list all publications that addressed similar research questions in the same dataset and indicate where you cited them in your paper
Reference: #1 —Jin R, Zhang S, Xiong J, Liu B. Global, regional and national burden of drug use disorders, 1990-2021: decomposition analysis, health inequality analysis and predictions to 2035. *Front Public Health*. 2025;13:1588607. doi:10.3389/fpubh.2025.1588607<br>Reference: #2 —Zhou J, He M, Zhou G, Yang T, Wang R, Li X. Global trends and inequalities in the burden of drug use disorders: a comprehensive analysis from 1990 to 2021 with future projections. *Front Public Health*. 2025;13:1655575. doi:10.3389/fpubh.2025.1655575<br>Reference: #3 —Danpanichkul P, Duangsonk K, Díaz LA, Chen VL, Rangan P, Sukphutanan B, et al. The burden of alcohol and substance use disorders in adolescents and young adults. *Drug Alcohol Depend*. 2025;266:112495. doi:10.1016/j.drugalcdep.2024.112495<br>Where cited: Introduction<br>Reference: #8 —GBD 2021 Diseases and Injuries Collaborators. Global incidence, prevalence, years lived with disability (YLDs), disability-adjusted life-years (DALYs), and healthy life expectancy (HALE) for 371 diseases and injuries in 204 countries and territories and 811 subnational locations, 1990-2021: a systematic analysis for the Global Burden of Disease Study 2021. *Lancet*. 2024;403(10440):2133-2161. doi:10.1016/S0140-6736(24)00757-8<br>Where cited: Matreial<br>Reference: #10 —GBD 2019 Diseases and Injuries Collaborators. Global burden of 369 diseases and injuries in 204 countries and territories, 1990-2019: a systematic analysis for the Global Burden of Disease Study 2019. Lancet. 2020;396(10258):1204-1222. doi:10.1016/S0140-6736(20)30925-9<br>Reference: #11 —Global Burden of Disease Collaborative Network. Global Burden of Disease Study 2021 (GBD 2021) Socio-Demographic Index (SDI) 1950 – 2021. Seattle, United States of America: Institute for Health Metrics and Evaluation (IHME), 2024. doi:10.6069/dwqg-3z75<br>Where cited: Matreial,Discussion
4. Please explain how you addressed multiple testing through an appropriately rigorous statistical threshold and indicate this in the methods section
Our analyses were designed primarily as descriptive epidemiological comparisons using GBD 2021 draw-based uncertainty intervals rather than hypothesis-testing exercises. Accordingly, we did not conduct large-scale significance testing across the many country–sex–age–year strata, and we did not interpret isolated p-values as confirmatory evidence. Where correlation analyses were reported, we treated p-values as descriptive only and emphasised effect sizes and uncertainty. To minimise the risk of spurious inferences arising from multiple comparisons, we (i) prespecified the principal outcomes (ASIR, ASPR, ASDR) and the primary stratification dimensions (age, sex, SDI, location); (ii) focused interpretation on consistent patterns observed across outcomes and strata rather than single comparisons; and (iii) avoided claims of “statistically significant” differences based solely on overlapping or non-overlapping uncertainty intervals.
5. Please declare to what extent have AI chatbots been used in developing your paper and to which parts of the paper did they contribute
No AI chatbots or generative AI tools were used in drafting, editing, analysis, interpretation, or figure/table preparation for this manuscript; all work was conducted by the authors.

Table S2. The incidence counts and age-standardised incidence rates of substance use disorders, by region, 1990 and 2021.

Region	1990		2021		Percentage change in ASRs
	Number (95%UI)	ASRs per 100000 (95%UI)	Number (95%UI)	ASRs per 100000 (95%UI)	
Global	52364456 (44759924–59912684)	1019.88 (880.38–1166.19)	69389100 (60494875–78767497)	843.37 (736.21–956.19)	-17.3 (-19.7–15.2)
Andean Latin America	318727 (275078–367890)	976.43 (840.85–1111.45)	514365 (438657–591255)	751.27 (644.74–860.13)	-23.1 (-27.5–18.7)
Australasia	294507 (257891–333131)	1366.63 (1197.37–1546.03)	476328 (418215–535847)	1498.32 (1314.14–1695.85)	9.6 (1.3–18.3)
Caribbean	383748 (327409–439713)	1145.42 (987.86–1300.20)	532781 (465681–600327)	1065.16 (928.28–1205.52)	-7.0 (-11.7–0.9)
Central Asia	1074950 (931838–1226182)	1722.02 (1502.56–1944.39)	1429556 (1244296–1619580)	1444.64 (1262.58–1637.38)	-16.1 (-21.4–10.8)
Central Europe	2086439 (1804492–2322819)	1544.45 (1342.05–1718.24)	1918981 (1681763–2149834)	1411.27 (1240.39–1585.28)	-8.6 (-11.7–5.3)
Central Latin America	1904757 (1620379–2190823)	1344.80 (1153.85–1532.92)	3072297 (2669677–3457218)	1148.54 (998.30–1291.81)	-14.6 (-17.8–11.5)
Central Sub-Saharan Africa	279123 (230227–328827)	680.10 (573.64–790.88)	733864 (613188–866792)	674.70 (566.69–786.44)	-0.8 (-8.9–7.0)
East Asia	10790412 (9262820–12442136)	826.81 (705.55–945.42)	12231292 (10373790–14109388)	754.70 (643.55–868.64)	-8.7 (-12.1–5.3)
Eastern Europe	4929596 (4141366–5765023)	1998.22 (1689.99–2339.41)	4604524 (3903161–5316717)	1910.57 (1644.48–2182.05)	-4.4 (-8.9–0.2)
Eastern Sub-Saharan Africa	1212992 (1014067–1413855)	893.36 (746.42–1035.44)	2949964 (2498822–3416313)	862.08 (730.27–987.12)	-3.5 (-7.0–0.9)
High-income Asia Pacific	1381465 (1190055–1586549)	732.74 (629.78–847.17)	1307026 (1137027–1501080)	688.47 (595.96–790.09)	-6.0 (-12.8–1.0)
High-income North America	4901472 (4168857–5655923)	1639.62 (1400.55–1900.56)	5704809 (5024862–6423972)	1558.92 (1371.83–1769.94)	-4.9 (-10.4–0.1)
North Africa and Middle East	1029444 (883865–1200368)	345.78 (296.43–401.54)	2233600 (1924536–2571957)	342.76 (297.59–392.70)	-0.9 (-2.9–1.3)
Oceania	35228 (29925–40889)	605.95 (514.25–702.58)	82590 (70686–97510)	624.29 (537.19–726.50)	3.0 (-5.0–11.9)
South Asia	9499750 (7859926–11147334)	1016.14 (835.73–1187.03)	14684032 (12616600–16941895)	775.93 (668.74–892.45)	-23.6 (-27.4–19.2)
Southeast Asia	2389207 (2045566–2761931)	545.86 (468.72–629.10)	3930259 (3406403–4463118)	524.17 (456.07–593.73)	-4.0 (-7.2–0.5)
Southern Latin America	656576 (558005–769409)	1348.68 (1149.43–1576.76)	927471 (785462–1087366)	1282.69 (1088.63–1510.75)	-4.9 (-12.1–4.5)
Southern Sub-Saharan Africa	563011 (466684–650465)	1269.68 (1047.90–1485.28)	942635 (789702–1100568)	1151.38 (967.26–1331.15)	-9.3 (-13.1–5.8)
Tropical Latin America	2401373 (2001418–2832066)	1656.95 (1380.41–1940.19)	3687156 (3118574–4236023)	1457.53 (1235.83–1668.43)	-12.0 (-18.3–5.8)
Western Europe	5703766 (5042360–6444924)	1403.12 (1232.79–1595.92)	6066021 (5478128–6725047)	1385.17 (1225.84–1555.40)	-1.3 (-5.4–2.2)

Western Sub-Saharan Africa	527914 (452210–607705)	360.22 (311.12–409.19)	1359548 (1174441–1552740)	355.97 (311.39–400.84)	-1.2 (-3.8–1.2)

Abbreviation: UI, uncertainty intervals; ASR, age-standardised rate.

Table S3. The DALYs counts and age-standardised DALY rates of substance use disorders, by region, 1990 and 2021.

Region	1990		2021		Percentage change in ASRs
	Number (95%UI)	ASRs per 100000 (95%UI)	Number (95%UI)	ASRs per 100000 (95%UI)	
Global	21900847 (17819199–26425326)	432.55 (353.13–521.34)	32543146 (26828986–38776444)	393.36 (323.56–469.03)	-9.1 (-11.9–5.7)
Andean Latin America	117655 (93482–144456)	382.47 (307.46–466.42)	169732 (135292–210729)	248.26 (198.09–307.21)	-35.1 (-41.3–27.8)
Australasia	127341 (102385–154573)	584.57 (469.53–710.08)	230739 (188777–277273)	711.30 (576.83–852.48)	21.7 (14.3–29.6)
Caribbean	121265 (94462–150735)	373.15 (291.41–461.38)	182020 (146897–226815)	358.06 (287.66–447.16)	-4.0 (-9.5–2.2)
Central Asia	408157 (325438–497288)	658.29 (529.31–796.44)	527920 (421054–650560)	531.08 (423.34–654.31)	-19.3 (-23.9–14.3)
Central Europe	767102 (636595–923638)	562.27 (464.38–678.52)	752218 (628281–905229)	528.04 (438.44–639.32)	-6.1 (-9.4–2.6)
Central Latin America	765666 (645357–914471)	584.69 (499.76–688.96)	994146 (796143–1236039)	371.26 (297.46–461.58)	-36.5 (-41.2–32.0)
Central Sub-Saharan Africa	91424 (70660–113170)	237.44 (183.47–291.47)	237158 (184857–302885)	228.32 (179.54–287.53)	-3.8 (-15.0–8.2)
East Asia	5612881 (4435091–6766175)	429.56 (342.30–516.80)	4478609 (3453232–5559499)	274.53 (210.41–341.20)	-36.1 (-42.9–30.0)
Eastern Europe	3055980 (2626226–3524317)	1225.08 (1052.49–1416.17)	2854366 (2475780–3268295)	1161.47 (1005.12–1334.44)	-5.2 (-9.8–0.7)
Eastern Sub-Saharan Africa	363440 (283261–457381)	299.49 (236.80–371.07)	865041 (678372–1100822)	279.35 (219.93–350.61)	-6.7 (-12.8–1.5)
High-income Asia Pacific	456152 (342835–590171)	239.60 (180.62–310.22)	429366 (327018–555499)	214.29 (161.87–279.50)	-10.6 (-16.2–5.0)
High-income North America	2197427 (1734037–2698796)	716.44 (565.30–883.23)	8306059 (7083721–9522436)	2193.48 (1861.45–2529.02)	206.2 (175.6–242.1)
North Africa and Middle East	593450 (467964–723201)	198.76 (158.66–240.27)	1332905 (1056565–1605485)	204.75 (162.28–246.00)	3.0 (-2.7–8.9)
Oceania	10985 (8442–13976)	196.11 (149.78–247.92)	24757 (18910–31526)	190.47 (146.41–242.68)	-2.9 (-11.8–7.2)
South Asia	2723676 (2083745–3413139)	301.31 (230.31–378.43)	4756070 (3693548–5884286)	254.49 (198.00–314.27)	-15.5 (-21.2–7.8)
Southeast Asia	918422 (711704–1131332)	220.56 (171.49–270.89)	1496053 (1173877–1851092)	198.63 (156.09–246.33)	-9.9 (-20.8–3.0)
Southern Latin America	215273 (166830–275456)	446.22 (346.77–569.99)	257819 (191001–337210)	351.60 (259.62–461.41)	-21.2 (-26.7–15.5)
Southern Sub-Saharan Africa	194409 (150139–242512)	449.62 (351.05–559.67)	301334 (238481–374979)	377.69 (301.10–469.71)	-16.0 (-19.8–11.9)
Tropical Latin America	749287 (589622–943047)	536.63 (427.13–672.81)	1275944 (1021407–1574629)	501.22 (400.12–617.70)	-6.6 (-10.2–2.3)
Western Europe	2216996 (1801379–2699684)	528.99 (428.24–647.25)	2587699 (2124293–3102656)	556.80 (451.53–671.59)	5.3 (2.8–8.0)
Western Sub-Saharan Africa	193860 (153325–238044)	142.95 (113.83–173.89)	483191 (381792–605859)	136.22 (108.18–170.26)	-4.7 (-14.1–7.4)

Abbreviation: UI, uncertainty intervals; ASR, age-standardised rate; DALY, Disability-Adjusted Life Year.

Table S4. The prevalence counts and age-standardised prevalence rates of alcohol use disorders, by region, 1990 and 2021.

Region	1990		2021		Percentage change in ASRs
	Number (95%UI)	ASRs per 100000 (95%UI)	Number (95%UI)	ASRs per 100000 (95%UI)	
Global	84545234 (72536872–98399854)	1697.90 (1459.91–1949.22)	111123390 (96356480–127901625)	1335.43 (1153.65–1539.75)	-21.3 (-23.5—19.1)
Andean Latin America	499677 (416602–594738)	1611.77 (1374.42–1861.54)	771301 (650143–904830)	1135.86 (966.25–1322.89)	-29.5 (-34.2—24.7)
Australasia	377970 (314962–448754)	1726.02 (1440.55–2061.10)	702638 (600260–818894)	2083.16 (1756.51–2450.41)	20.7 (8.7–33.8)
Caribbean	594118 (501435–702143)	1839.83 (1579.75–2140.95)	853725 (734976–979278)	1682.64 (1439.64–1935.14)	-8.5 (-13.4—3.1)
Central Asia	1954305 (1649825–2301972)	3219.26 (2735.24–3696.54)	2543285 (2223653–2936758)	2581.06 (2252.39–2969.55)	-19.8 (-25.6—12.1)
Central Europe	3680908 (3246226–4100862)	2670.87 (2350.02–2991.64)	3516462 (3152920–3930826)	2393.52 (2115.79–2716.10)	-10.4 (-13.8—6.8)
Central Latin America	3130472 (2659358–3679548)	2311.96 (1991.21–2644.99)	5098780 (4395564–5808895)	1907.84 (1643.23–2172.87)	-17.5 (-20.6—13.9)
Central Sub-Saharan Africa	426636 (351554–511651)	1107.49 (930.84–1302.36)	1097207 (896283–1323700)	1075.46 (895.54–1258.10)	-2.9 (-10.4–5.8)
East Asia	15391565 (12843054–18319981)	1224.59 (1030.19–1431.89)	19820498 (16985328–23214405)	1155.35 (971.48–1350.39)	-5.7 (-9.9—1.4)
Eastern Europe	9957529 (8733084–11390068)	3907.46 (3410.91–4449.14)	8603156 (7635147–9759598)	3292.73 (2901.33–3724.07)	-15.7 (-19.4—12.0)
Eastern Sub-Saharan Africa	2025735 (1703270–2404834)	1609.35 (1377.73–1863.31)	4803137 (4052397–5682782)	1509.56 (1297.80–1731.17)	-6.2 (-10.0—2.2)
High-income Asia Pacific	2253693 (1909879–2677447)	1177.47 (997.88–1408.56)	2291329 (1963986–2600782)	1054.43 (890.89–1241.51)	-10.4 (-20.1—1.8)
High-income North America	8231443 (7042749–9674659)	2686.09 (2297.68–3156.57)	8545847 (7389597–9816603)	2155.75 (1849.51–2484.03)	-19.7 (-24.1—15.3)
North Africa and Middle East	1157374 (930197–1429175)	411.53 (335.30–502.79)	2435486 (1943712–2983132)	381.90 (311.10–465.20)	-7.2 (-9.9—4.4)
Oceania	43792 (35533–52433)	822.70 (687.28–970.81)	107220 (86971–128610)	855.34 (706.06–1016.90)	4.0 (-6.7–16.4)
South Asia	15489044 (12807037–18420779)	1731.61 (1456.30–2040.45)	22905959 (19785466–26676135)	1244.56 (1077.22–1441.41)	-28.1 (-31.9—24.4)
Southeast Asia	3304517 (2739988–3960792)	805.52 (679.47–943.36)	5614233 (4793111–6524032)	747.43 (637.59–866.79)	-7.2 (-10.7—3.3)
Southern Latin America	1170898 (974528–1406046)	2428.19 (2025.85–2915.68)	1556760 (1287426–1836506)	2113.45 (1736.46–2517.42)	-13.0 (-19.9—4.3)
Southern Sub-Saharan Africa	919192 (768656–1078549)	2207.57 (1882.68–2553.06)	1558757 (1322133–1831018)	1956.25 (1686.58–2285.51)	-11.4 (-15.0—7.7)

Tropical Latin America	3979422 (3337272–4664516)	2869.40 (2431.73–3331.75)	6521023 (5654586–7471964)	2539.00 (2208.26–2904.37)	-11.5 (-17.3—4.9)
Western Europe	9221919 (7990618–10654464)	2193.21 (1892.94–2557.62)	9957985 (8743116–11242354)	2113.94 (1832.43–2466.96)	-3.6 (-7.5–0.4)
Western Sub-Saharan Africa	735024 (615765–875745)	531.18 (450.68–621.17)	1818603 (1514011–2157564)	511.90 (434.14–593.59)	-3.6 (-6.0—1.0)

Abbreviation: UI, uncertainty intervals; ASR, age-standardised rate.

Table S5. The incidence counts and age-standardised incidence rates of alcohol use disorders, by region, 1990 and 2021.

Region	1990		2021		Percentage change in ASRs
	Number (95%UI)	ASRs per 100000 (95%UI)	Number (95%UI)	ASRs per 100000 (95%UI)	
Global	42320999 (34758376–49270544)	835.57 (688.06–971.83)	55779737 (46555775–64314073)	673.98 (563.13–776.68)	-19.3 (-21.9—16.9)
Andean Latin America	265129 (221740–311394)	831.73 (701.94–959.47)	412109 (339349–484587)	604.03 (502.02–708.93)	-27.4 (-32.3—22.6)
Australasia	194198 (158018–230107)	889.48 (723.88–1054.87)	352605 (303510–409376)	1072.84 (906.15–1254.21)	20.6 (7.9–33.8)
Caribbean	317095 (261177–372749)	964.77 (807.14–1114.06)	446330 (375548–511593)	885.07 (741.85–1017.77)	-8.3 (-13.7—1.1)
Central Asia	962340 (820098–1109416)	1556.22 (1345.68–1768.37)	1263676 (1085644–1449520)	1274.92 (1102.99–1463.29)	-18.1 (-23.7—12.2)
Central Europe	1865022 (1591996–2089024)	1368.60 (1164.44–1531.63)	1724043 (1488090–1952587)	1227.02 (1054.53–1395.00)	-10.3 (-13.8—6.7)
Central Latin America	1678312 (1408528–1961229)	1203.48 (1009.28–1387.94)	2687853 (2296817–3063178)	1004.50 (858.51–1143.59)	-16.5 (-20.0—13.1)
Central Sub-Saharan Africa	228363 (182837–277249)	572.54 (465.65–678.12)	595511 (481290–723017)	564.65 (462.74–674.22)	-1.4 (-10.9–8.0)
East Asia	7776827 (6246679–9318005)	608.67 (488.11–724.36)	9677793 (7816914–11502865)	580.77 (468.22–688.35)	-4.6 (-9.0–0.3)
Eastern Europe	4337052 (3580306–5184927)	1735.86 (1438.56–2071.55)	4074748 (3384440–4793312)	1634.85 (1373.51–1906.75)	-5.8 (-10.7–1.3)
Eastern Sub-Saharan Africa	1045327 (841316–1250574)	794.42 (652.55–929.76)	2532002 (2079124–3011961)	760.99 (634.43–884.31)	-4.2 (-8.0–0.6)
High-income Asia Pacific	993349 (816512–1171944)	523.98 (430.62–620.79)	982129 (813986–1142661)	484.09 (395.56–572.37)	-7.6 (-16.8–2.6)
High-income North America	3879681 (3186222–4596304)	1273.04 (1046.34–1516.15)	3991101 (3341509–4658925)	1038.85 (865.87–1222.59)	-18.4 (-23.9—13.5)
North Africa and Middle East	610848 (481775–758354)	211.66 (167.64–257.75)	1283425 (1010585–1556661)	199.24 (157.49–240.04)	-5.9 (-8.4–2.9)
Oceania	23771 (18851–28963)	433.76 (356.46–520.09)	57778 (46803–70062)	451.04 (368.95–542.32)	4.0 (-7.2–16.0)
South Asia	8260098 (6589384–9824079)	895.15 (719.75–1060.85)	12086451 (10010684–14185859)	644.52 (536.38–756.74)	-28.0 (-31.9—23.7)
Southeast Asia	1730082 (1395514–2094240)	408.52 (335.70–488.50)	2880542 (2369407–3369337)	382.69 (315.23–447.80)	-6.3 (-10.1–2.0)
Southern Latin America	564095 (465713–678104)	1162.73 (962.17–1395.36)	792323 (651337–953324)	1086.56 (885.56–1316.53)	-6.6 (-14.7–4.3)
Southern Sub-Saharan Africa	480556 (390199–568105)	1108.10 (895.22–1318.86)	806281 (655033–963411)	989.87 (810.26–1166.90)	-10.7 (-14.9—6.9)
Tropical Latin America	2116042 (1718081–2532076)	1482.27 (1205.95–1765.44)	3264931 (2716040–3806627)	1277.13 (1065.61–1490.95)	-13.8 (-20.6—7.2)
Western Europe	4607134 (3952105–5320230)	1112.62 (947.88–1295.15)	4907355 (4318957–5508126)	1083.17 (923.39–1247.99)	-2.6 (-7.5–1.7)
Western Sub-Saharan Africa	385678 (312390–460490)	271.71 (223.61–317.83)	960751 (776028–1143594)	261.28 (216.36–302.72)	-3.8 (-6.7–0.8)

Abbreviation: UI, uncertainty intervals; ASR, age-standardised rate.

Table S6. The DALYs counts and age-standardised DALY rates of alcohol use disorders, by region, 1990 and 2021.

Region	1990		2021		Percentage change in ASRs
	Number (95%UI)	ASRs per 100000 (95%UI)	Number (95%UI)	ASRs per 100000 (95%UI)	
Global	12990244 (10296142–16452164)	266.11 (212.48–335.00)	16980984 (13531791–21197118)	202.39 (161.07–253.23)	-23.9 (-27.5—20.6)
Andean Latin America	87113 (68736–109397)	299.45 (238.93–369.09)	105371 (81695–137195)	156.67 (122.30–203.21)	-47.7 (-53.8—39.8)
Australasia	48185 (36005–64151)	219.34 (163.34–293.26)	85023 (62672–112767)	247.06 (179.75–333.41)	12.6 (2.5–23.3)
Caribbean	88111 (68427–112631)	282.39 (221.52–355.31)	136608 (108216–173632)	265.76 (210.15–338.54)	-5.9 (-12.2–1.4)
Central Asia	315789 (253478–387395)	522.55 (420.50–638.80)	369342 (294110–466345)	372.31 (295.99–471.26)	-28.8 (-34.0—22.9)
Central Europe	642907 (533523–783025)	463.99 (383.42–565.02)	628084 (529248–763703)	414.25 (345.92–507.31)	-10.7 (-14.4—7.0)
Central Latin America	626452 (524543–756969)	497.81 (423.07–587.21)	756667 (597304–960164)	282.86 (223.45–358.96)	-43.2 (-48.4—38.3)
Central Sub-Saharan Africa	67663 (50324–86452)	184.67 (138.80–233.29)	166553 (126456–216627)	169.05 (129.26–216.13)	-8.5 (-21.0–6.1)
East Asia	1998494 (1471330–2649509)	161.07 (119.05–211.85)	2742503 (2038789–3517759)	157.30 (116.89–203.38)	-2.3 (-15.8–7.9)
Eastern Europe	2287646 (1982214–2644738)	901.48 (780.08–1044.20)	2020286 (1735975–2358012)	758.34 (649.65–889.05)	-15.9 (-20.1—11.6)
Eastern Sub-Saharan Africa	276471 (210389–357570)	238.66 (183.24–300.56)	624521 (466963–833600)	212.77 (162.90–274.94)	-10.8 (-18.0—1.0)
High-income Asia Pacific	289326 (213247–387551)	149.73 (109.32–202.08)	276741 (207429–372868)	124.23 (90.96–171.68)	-17.0 (-25.9—8.7)
High-income North America	1119388 (852950–1457865)	364.39 (277.50–475.30)	1498596 (1241443–1822680)	357.14 (290.09–438.94)	-2.0 (-8.3–5.1)
North Africa and Middle East	139769 (98929–190448)	50.03 (36.14–67.55)	273408 (192327–377908)	42.80 (29.88–59.00)	-14.5 (-20.0—8.6)
Oceania	6422 (4583–8580)	124.78 (89.75–165.05)	15077 (11166–19998)	121.66 (91.17–161.14)	-2.5 (-15.3–11.9)
South Asia	2036766 (1508102–2651374)	230.20 (170.28–299.53)	3234752 (2468306–4267367)	175.81 (133.79–230.88)	-23.6 (-29.6—13.8)
Southeast Asia	594046 (447722–744719)	152.27 (115.30–190.45)	968818 (756255–1251761)	127.42 (99.39–164.81)	-16.3 (-29.8–0.9)
Southern Latin America	163580 (124994–212322)	341.70 (261.95–443.30)	179376 (129377–243000)	241.31 (172.11–327.20)	-29.4 (-36.6—21.9)
Southern Sub-Saharan Africa	111731 (82348–149257)	274.19 (201.88–364.35)	184481 (135488–244366)	234.40 (173.38–306.81)	-14.5 (-18.9—10.3)
Tropical Latin America	587225 (460604–743755)	434.54 (343.57–545.23)	963388 (766472–1211494)	371.56 (294.37–468.70)	-14.5 (-18.4—10.2)
Western Europe	1369765 (1081261–1746034)	317.65 (247.88–410.04)	1424677 (1126669–1808175)	280.44 (212.47–368.53)	-11.7 (-15.5–8.2)

Western Sub-Saharan Africa	133395	(105244–168990)	105.05	(82.37–129.75)	326709	(252805–418709)	98.95	(76.91–126.05)	-5.8	(-17.6–11.0)

Abbreviation: UI, uncertainty intervals; ASR, age-standardised rate; DALY, disability-adjusted life year.

Table S7. **The prevalence counts and age-standardised prevalence rates of drug use disorders, by region, 1990 and 2021.**

Region	1990		2021		Percentage change in ASRs
	**Number (95%UI)**	**ASRs per 100000 (95%UI)**	**Number (95%UI)**	**ASRs per 100000 (95%UI)**	
Global	39620619 (34071985–46420659)	709.15 (618.81–824.55)	53115936 (46999805–60949054)	663.80 (584.52–766.14)	-6.4 (-9.6—3.4)
Andean Latin America	184911 (153484–224207)	477.19 (404.11–573.00)	339995 (286940–407832)	480.27 (406.37–576.63)	0.6 (-4.2–5.2)
Australasia	476382 (426796–541882)	2231.60 (1996.93–2541.13)	530217 (478541–594230)	1819.35 (1632.77–2054.71)	-18.5 (-23.3—13.0)
Caribbean	279260 (216019–366665)	727.81 (576.21–930.60)	355323 (277254–455909)	733.78 (568.30–946.83)	0.8 (-2.9–4.6)
Central Asia	389627 (322331–477058)	549.62 (461.22–662.76)	563160 (480004–667119)	574.49 (483.62–687.87)	4.5 (1.6–8.0)
Central Europe	773851 (645713–928215)	631.15 (523.55–762.89)	644902 (569859–745989)	662.66 (571.49–776.46)	5.0 (-0.6–11.0)
Central Latin America	823981 (699123–993826)	489.62 (422.16–578.33)	1420514 (1237211–1635360)	529.59 (460.91–609.71)	8.2 (4.1–12.6)
Central Sub-Saharan Africa	149823 (114081–202618)	297.75 (238.08–387.13)	403027 (309609–542841)	306.32 (246.24–397.68)	2.9 (1.4–4.5)
East Asia	11677286 (9927237–13860436)	810.69 (697.85–954.84)	8012070 (6895063–9433061)	589.83 (494.67–703.92)	-27.2 (-31.5—22.0)
Eastern Europe	2199137 (1915744–2525301)	963.68 (829.48–1114.46)	1935374 (1732723–2177485)	1041.24 (908.44–1198.50)	8.0 (3.9–12.0)
Eastern Sub-Saharan Africa	580466 (440470–776486)	327.74 (260.28–419.69)	1411568 (1067354–1896498)	325.17 (257.73–422.17)	-0.8 (-3.8–1.4)
High-income Asia Pacific	1466311 (1220530–1868929)	798.76 (660.32–1022.92)	1203527 (1028637–1489142)	781.29 (644.13–995.33)	-2.2 (-4.4–0.5)
High-income North America	5833558 (5056469–6765849)	1997.89 (1722.10–2324.30)	12918134 (11781272–14224634)	3668.00 (3323.49–4067.36)	83.6 (66.9–103.3)
North Africa and Middle East	1233270 (1038866–1459417)	378.78 (326.81–439.00)	2771197 (2422239–3187680)	422.68 (369.99–485.49)	11.6 (8.1–15.6)
Oceania	46273 (32921–64390)	668.94 (495.56–902.66)	98706 (72689–133720)	672.72 (503.93–893.96)	0.6 (-1.4–3.2)
South Asia	3970038 (3191740–5120145)	380.92 (311.84–479.25)	7844211 (6513450–9819055)	391.33 (327.33–483.68)	2.7 (-2.4–7.8)
Southeast Asia	2604146 (2060760–3282541)	519.61 (419.67–644.87)	3872469 (3139748–4776982)	524.30 (424.34–649.16)	0.9 (-1.8–3.8)
Southern Latin America	365413 (319153–423108)	729.13 (636.60–843.35)	565191 (505941–640497)	815.88 (730.49–926.72)	11.9 (5.1–19.2)
Southern Sub-Saharan Africa	344062 (294204–409546)	670.31 (586.96–776.34)	541072 (455101–657468)	639.35 (539.98–771.88)	-4.6 (-9.4–1.2)
Tropical Latin America	1477411 (1185378–1860113)	903.62 (741.68–1122.15)	2099771 (1788694–2510048)	888.21 (750.00–1066.61)	-1.7 (-8.6–5.5)
Western Europe	4345994 (3823480–4942184)	1126.22 (986.69–1287.06)	4519737 (4106574–5029749)	1201.17 (1081.17–1351.18)	6.7 (2.4–11.2)
Western Sub-Saharan Africa	399419 (321303–504249)	234.19 (195.34–284.58)	1065774 (875503–1332478)	236.65 (199.54–285.92)	1.0 (-1.6–3.7)

Abbreviation: UI, uncertainty intervals; ASR, age-standardised rate.

Table S8. The incidence counts and age-standardised incidence rates of drug use disorders, by region, 1990 and 2021.

Region	1990		2021		Percentage change in ASRs
	Number (95%UI)	ASRs per 100000 (95%UI)	Number (95%UI)	ASRs per 100000 (95%UI)	
Global	10043456 (8541086–11526400)	184.31 (156.91–211.67)	13609362 (11625288–15667184)	169.39 (145.14–195.01)	-8.1 (-10.3—6.0)
Andean Latin America	53597 (45091–62341)	144.70 (120.69–169.42)	102256 (86120–118552)	147.25 (123.64–171.11)	1.8 (-2.0–5.9)
Australasia	100309 (87560–117259)	477.15 (415.54–555.65)	123723 (106675–141156)	425.48 (369.39–483.04)	-10.8 (-19.1—2.8)
Caribbean	66652 (54650–81485)	180.65 (149.44–218.09)	86450 (70519–104744)	180.09 (147.22–220.40)	-0.3 (-4.1–3.7)
Central Asia	112610 (94819–133624)	165.80 (140.28–195.03)	165880 (140144–194129)	169.72 (143.57–197.09)	2.4 (-0.7–6.5)
Central Europe	221417 (184626–261255)	175.85 (147.21–208.17)	194939 (163007–228660)	184.25 (155.27–214.63)	4.8 (0.9–8.2)
Central Latin America	226444 (191217–265255)	141.32 (118.00–165.85)	384444 (323943–446656)	144.04 (121.38–167.38)	1.9 (-0.3–4.7)
Central Sub-Saharan Africa	50760 (41525–61585)	107.56 (89.53–127.51)	138352 (114529–166974)	110.05 (91.77–129.82)	2.3 (-1.5–6.6)
East Asia	3013584 (2546149–3494624)	218.15 (184.94–253.87)	2553499 (2134778–3029328)	173.93 (146.09–204.63)	-20.3 (-24.2—16.3)
Eastern Europe	592543 (506284–679474)	262.36 (224.87–300.18)	529776 (457124–607120)	275.72 (238.80–312.90)	5.1 (1.4–9.3)
Eastern Sub-Saharan Africa	167665 (135071–206477)	98.94 (81.32–117.53)	417962 (340922–513396)	101.09 (83.77–119.60)	2.2 (-0.7–4.9)
High-income Asia Pacific	388116 (318787–468376)	208.76 (170.85–253.22)	324897 (268440–387745)	204.38 (168.19–247.27)	-2.1 (-4.5–0.5)
High-income North America	1021791 (877231–1180896)	366.58 (312.82–426.29)	1713708 (1503720–1941996)	520.07 (454.13–592.82)	41.9 (32.7–52.0)
North Africa and Middle East	418595 (350340–495004)	134.12 (112.41–158.09)	950175 (796662–1121368)	143.52 (120.87–169.07)	7.0 (4.7–9.8)
Oceania	11458 (9045–14625)	172.19 (139.24–212.00)	24812 (20002–30730)	173.25 (141.60–212.00)	0.6 (-2.9–3.7)
South Asia	1239652 (1023324–1479405)	120.99 (100.92–142.05)	2597580 (2168941–3042955)	131.41 (109.78–153.28)	8.6 (5.0–12.1)
Southeast Asia	659125 (543435–779425)	137.34 (113.22–161.97)	1049717 (869354–1231733)	141.48 (116.93–166.01)	3.0 (1.1–5.2)
Southern Latin America	92482 (77702–108917)	185.95 (156.40–219.07)	135148 (114722–157009)	196.13 (167.54–227.34)	5.5 (0.5–11.1)
Southern Sub-Saharan Africa	82455 (71110–96529)	161.58 (139.28–185.37)	136354 (115592–157588)	161.51 (137.31–186.47)	0.0 (-3.5–3.7)
Tropical Latin America	285331 (239090–346022)	174.68 (146.99–208.86)	422226 (359194–486315)	180.40 (153.43–207.80)	3.3 (-2.8–9.4)
Western Europe	1096632 (932291–1271153)	290.50 (248.87–336.73)	1158667 (1007513–1338089)	302.00 (262.87–348.16)	4.0 (-1.1–9.2)
Western Sub-Saharan Africa	142236 (117504–169016)	88.51 (72.58–106.10)	398798 (333659–469311)	94.68 (79.60–111.36)	7.0 (3.3–11.8)

Abbreviation: UI, uncertainty intervals; ASR, age-standardised rate.

Table S9. The DALYs counts and age-standardised DALY rates of drug use disorders, by region, 1990 and 2021.

Region	1990		2021		Percentage change in ASRs
	Number (95%UI)	ASRs per 100000 (95%UI)	Number (95%UI)	ASRs per 100000 (95%UI)	
Global	8910603 (7055603–10630912)	166.44 (132.55–198.40)	15562162 (12752222–18119264)	190.97 (156.11–222.79)	14.7 (9.2–21.1)
Andean Latin America	30542 (22397–38683)	83.02 (62.22–104.32)	64360 (50048–80102)	91.59 (71.54–113.89)	10.3 (1.3–21.0)
Australasia	79157 (63933–93224)	365.23 (294.85–430.61)	145716 (122596–167902)	464.24 (387.42–539.76)	27.1 (17.2–38.5)
Caribbean	33153 (23763–42405)	90.76 (65.78–116.08)	45412 (34712–57360)	92.30 (70.37–117.00)	1.7 (-4.0–9.4)
Central Asia	92369 (67553–118438)	135.73 (100.13–171.36)	158578 (121995–192700)	158.77 (122.14–192.69)	17.0 (8.8–27.6)
Central Europe	124195 (97749–151799)	98.28 (77.04–120.40)	124134 (100102–148754)	113.79 (90.96–137.08)	15.8 (10.8–21.6)
Central Latin America	139214 (105074–176665)	86.88 (65.99–109.39)	237479 (184801–294878)	88.41 (68.83–109.78)	1.8 (-2.3–6.8)
Central Sub-Saharan Africa	23761 (17784–30833)	52.77 (39.86–67.88)	70605 (51500–90811)	59.27 (43.57–75.48)	12.3 (0.6–26.9)
East Asia	3614387 (2897066–4299279)	268.49 (217.53–316.18)	1736106 (1342137–2120314)	117.23 (89.99–144.50)	-56.3 (-61.8—51.6)
Eastern Europe	768334 (614076–907539)	323.60 (257.79–382.26)	834081 (709962–965281)	403.12 (337.84–468.51)	24.6 (16.1–34.6)
Eastern Sub-Saharan Africa	86969 (66611–111946)	60.83 (47.07–78.07)	240519 (186125–298671)	66.58 (51.76–82.03)	9.5 (-2.2–20.5)
High-income Asia Pacific	166825 (118067–220263)	89.87 (63.71–119.15)	152624 (111896–194901)	90.07 (65.11–117.63)	0.2 (-3.3–4.3)
High-income North America	1078040 (831795–1308307)	352.05 (270.73–427.06)	6807463 (5764549–7844234)	1836.34 (1547.74–2122.45)	421.5 (372.2–483.4)
North Africa and Middle East	453681 (353056–564083)	148.73 (117.32–181.45)	1059497 (850242–1265837)	161.95 (129.92–193.35)	8.9 (0.6–16.7)
Oceania	4563 (3241–6143)	71.33 (51.57–93.20)	9680 (6943–12723)	68.81 (49.71–89.73)	-3.5 (-11.5–4.1)
South Asia	686910 (536015–854811)	71.11 (56.10–87.26)	1521317 (1197077–1867551)	78.68 (62.13–95.44)	10.7 (3.3–18.2)
Southeast Asia	324376 (232648–428994)	68.29 (50.24–88.66)	527235 (390182–674457)	71.21 (52.71–91.13)	4.3 (-1.6–11.1)
Southern Latin America	51693 (35510–68801)	104.52 (72.10–139.03)	78444 (56505–102080)	110.29 (79.08–143.92)	5.5 (-1.9–14.8)
Southern Sub-Saharan Africa	82678 (64118–100809)	175.43 (138.47–210.37)	116853 (95213–138212)	143.29 (117.00–169.05)	-18.3 (-23.5—12.3)
Tropical Latin America	162062 (110808–213561)	102.09 (71.29–133.35)	312556 (238402–385075)	129.66 (98.32–160.11)	27.0 (18.9–40.4)
Western Europe	847231 (685035–1005492)	211.34 (170.89–250.97)	1163022 (975084–1348151)	276.35 (230.25–322.25)	30.8 (26.8–35.5)
Western Sub-Saharan Africa	60464 (42964–79862)	37.90 (27.48–48.72)	156482 (110916–206265)	37.27 (26.73–48.09)	-1.7 (-4.9–1.8)

Abbreviation: UI, uncertainty intervals; ASR, age-standardised rate; DALY, disability-adjusted life year.

Table S10. The prevalence, incidence, and DALY counts and age-standardised rates of substance use disorders, by SDI region, 1990 and 2021.

SDI level	1990		2021		Percentage change in ASRs
	Number (95%UI)	ASRs per 100000 (95%UI)	Number (95%UI)	ASRs per 100000 (95%UI)	
**Prevalence**					
High SDI	31577844(28315256-34988589)	3340.60(2996.27-3716.07)	41307962(37871459-44816162)	3680.79(3356.60-4021.64)	10.2(6.5-14.7)
High-middle SDI	32107259(28657121-35562241)	2865.64(2564.45-3168.77)	32695530(29326877-36352442)	2237.28(1998.89-2490.42)	-21.9(-23.7--19.9)
Low SDI	6372574(5441789-7304376)	1698.98(1469.59-1934.89)	13629928(11767024-15545580)	1511.14(1320.37-1708.02)	-11.1(-13.9--8.3)
Low-middle SDI	18700042(16019645-21399944)	1935.07(1670.22-2206.02)	30143790(26488494-34033976)	1597.01(1411.15-1792.57)	-17.5(-20.3--14.5)
Middle SDI	34105532(29945059-38572436)	2027.23(1784.86-2277.79)	44936883(39868193-50424504)	1701.24(1508.13-1906.91)	-16.1(-18.4--13.5)
**Incidence**					
High SDI	12366750(10841797-14036965)	1310.75(1151.29-1487.89)	14233711(12689795-15753557)	1266.27(1124.38-1418.82)	-3.4(-6.7--0.3)
High-middle SDI	13404214(11555090-15254378)	1201.73(1029.82-1365.60)	14470816(12530348-16449435)	985.11(858.23-1113.71)	-18.0(-20.5--15.6)
Low SDI	3037593(2535728-3532520)	803.05(670.41-932.65)	6473355(5496822-7424666)	712.34(610.57-811.74)	-11.3(-14.6--7.9)
Low-middle SDI	8939816(7496023-10404446)	916.04(767.88-1063.03)	14211247(12315195-16242571)	748.66(645.85-858.69)	-18.3(-21.5--15.0)
Middle SDI	14557741(12326651-16826206)	875.68(747.55-1005.43)	19934670(17120505-22934469)	748.90(645.82-856.21)	-14.5(-17.0--11.8)
**DALYs**					
High SDI	5117327(4117020-6234632)	531.95(426.99-647.80)	11966362(10095922-13788052)	1031.45(863.20-1200.31)	93.9(79.3-110.4)
High-middle SDI	6445197(5329655-7657771)	578.02(479.00-685.74)	6101710(5004966-7314975)	405.70(331.69-488.39)	-29.8(-32.5--27.4)
Low SDI	910891(705478-1141882)	256.12(199.51-319.03)	2005521(1578111-2520672)	233.17(184.25-289.73)	-9.0(-14.3--2.0)
Low-middle SDI	2952934(2308098-3622889)	315.02(247.19-387.27)	5074962(4044887-6209573)	271.72(215.88-330.86)	-13.7(-18.9--7.5)
Middle SDI	6453622(5187608-7845726)	392.48(317.87-474.96)	7369859(5871863-9026378)	275.90(219.61-337.69)	-29.7(-34.1--25.9)

Abbreviation: UI, uncertainty intervals; ASR, age-standardised rate; SDI, socio-demographic index, DALYs, disability-adjusted life years.

Table S11. The prevalence, incidence, and DALY counts and age-standardised rates of alcohol use disorders, by SDI region, 1990 and 2021.

SDI level	1990		2021		Percentage change in ASRs
	Number (95%UI)	ASRs per 100000 (95%UI)	Number (95%UI)	ASRs per 100000 (95%UI)	
**Prevalence**					
High SDI	20326282 (17613187–23464281)	2107.66 (1824.14–2431.09)	22709307 (20087944–25557111)	1847.64 (1603.23–2115.44)	-12.3 (-15.4—9.6)
High-middle SDI	23265889 (20266228–26538507)	2111.15 (1845.60–2401.33)	24680302 (21625646–28155385)	1588.01 (1373.31–1829.90)	-24.8 (-27.1—22.3)
Low SDI	4890138 (4123371–5814331)	1369.80 (1174.81–1589.26)	10036617 (8444707–11810942)	1181.48 (1011.32–1369.74)	-13.7 (-17.1—10.6)
Low-middle SDI	14385002 (12071069–16936158)	1546.54 (1314.08–1798.53)	22241522 (19128712–25766409)	1208.59 (1048.55–1386.76)	-21.9 (-25.1—18.7)
Middle SDI	21581439 (17942411–25480174)	1364.51 (1148.04–1594.00)	31347460 (27022464–36250293)	1159.20 (992.08–1343.29)	-15.0 (-18.0—12.0)
**Incidence**					
High SDI	9823071 (8317879–11370057)	1026.10 (867.95–1191.65)	10838691 (9294474–12265432)	915.37 (772.84–1052.98)	-10.8 (-14.5—7.3)
High-middle SDI	10975208 (9135927–12759532)	988.52 (818.25–1148.75)	12005934 (9976133–13938712)	795.45 (667.73–918.44)	-19.5 (-22.2—16.7)
Low SDI	2570120 (2078709–3041287)	695.68 (567.28–817.69)	5312921 (4367982–6250152)	601.52 (500.85–695.22)	-13.5 (-17.2—9.9)
Low-middle SDI	7593608 (6145383–8956122)	791.29 (643.63–932.41)	11605238 (9682341–13531258)	618.10 (515.84–719.74)	-21.9 (-25.3—18.5)
Middle SDI	11309415 (9103569–13395977)	697.17 (563.03–821.40)	15962303 (13065603–18833790)	593.72 (488.60–695.56)	-14.8 (-17.8—11.6)
**DALYs**					
High SDI	3013577 (2367827–3830199)	309.62 (241.73–395.65)	3632790 (2970351–4483547)	278.84 (222.21–350.09)	-9.9 (-12.4––7.3)
High-middle SDI	4009313 (3274804–4947783)	366.70 (300.51–451.03)	4032112 (3275695–4945727)	251.78 (202.57–313.29)	-31.3 (-34.6—28.8)
Low SDI	666333 (498519–862827)	194.91 (146.02–246.93)	1359657 (1026256–1779664)	166.70 (128.40–214.96)	-14.5 (-20.4—6.1)
Low-middle SDI	2181148 (1654021–2777966)	240.18 (184.46–303.48)	3481427 (2756677–4461768)	190.44 (151.00–243.31)	-20.7 (-26.8—12.7)
Middle SDI	3103902 (2378767–4018676)	201.12 (154.13–255.91)	4457115 (3444399–5660991)	163.00 (125.67–208.35)	-19.0 (-25.0—13.9)

Abbreviation: UI, uncertainty intervals; ASR, age-standardised rate; SDI, socio-demographic index, DALYs, disability-adjusted life years.

Table S12. The prevalence, incidence, and DALY counts and age-standardised rates of drug use disorders, by SDI region, 1990 and 2021.

SDI level	1990 Number (95%UI)	1990 ASRs per 100000 (95%UI)	2021 Number (95%UI)	2021 ASRs per 100000 (95%UI)	Percentage change in ASRs
**Prevalence**					
High SDI	11716161 (10205716–13653997)	1282.80 (1114.26–1499.92)	19257655 (17598070–21398278)	1897.69 (1710.93–2137.33)	47.9 (38.9–58.7)
High-middle SDI	9136947 (7931599–10525728)	779.94 (678.13–894.16)	8255012 (7332624–9380594)	667.46 (584.49–771.03)	-14.4 (-16.8—11.8)
Low SDI	1512866 (1193635–1960808)	336.54 (276.98–420.79)	3654504 (2931631–4700261)	335.76 (278.03–416.83)	-0.2 (-2.1–1.7)
Low-middle SDI	4423813 (3635045–5541738)	398.90 (337.37–486.57)	8049659 (6825502–9817912)	395.86 (339.37–479.11)	-0.8 (-3.4–2.0)
Middle SDI	12797456 (10927813–15186456)	677.71 (589.08–788.40)	13859823 (11976148–16249385)	552.53 (475.41–653.60)	-18.5 (-22.9—14.0)
**Incidence**					
High SDI	2543678 (2151253–2950158)	284.65 (242.42–330.56)	3395020 (2984732–3860120)	350.90 (307.36–400.21)	23.3 (18.2–29.4)
High-middle SDI	2429006 (2073813–2807202)	213.21 (181.57–246.44)	2464882 (2080289–2880814)	189.65 (161.53–218.09)	-11.0 (-13.3—8.7)
Low SDI	467473 (388094–557289)	107.37 (88.96–125.35)	1160434 (968968–1371410)	110.82 (92.59–128.79)	3.2 (1.0–5.9)
Low-middle SDI	1346208 (1131389–1591129)	124.75 (105.84–145.45)	2606009 (2213505–3032279)	130.55 (110.96–151.41)	4.7 (2.0–7.4)
Middle SDI	3248326 (2786171–3721937)	178.51 (152.88–204.74)	3972367 (3365469–4603392)	155.19 (131.25–179.27)	-13.1 (-16.1—10.0)
**DALYs**					
High SDI	2103750 (1644036–2549563)	222.34 (173.15–269.45)	8333572 (7048827–9607579)	752.61 (630.61–872.87)	238.5 (210.7–272.5)
High-middle SDI	2435884 (1915886–2897939)	211.32 (166.86–251.33)	2069598 (1664133–2459504)	153.92 (122.22–185.21)	-27.2 (-31.1—23.9)
Low SDI	244558 (188284–309825)	61.20 (47.94–76.72)	645865 (497371–797895)	66.47 (51.71–81.05)	8.6 (2.7–15.3)
Low-middle SDI	771787 (605434–962184)	74.83 (59.16–91.85)	1593534 (1239893–1935327)	81.28 (63.74–98.29)	8.6 (3.5–13.9)
Middle SDI	3349720 (2667548–3959106)	191.36 (154.29–223.58)	2912744 (2307573–3479457)	112.90 (89.50–135.09)	-41.0 (-46.5—36.2)

Abbreviation: UI, uncertainty intervals; ASR, age-standardised rate; SDI, socio-demographic index; DALYs, disability-adjusted life years.

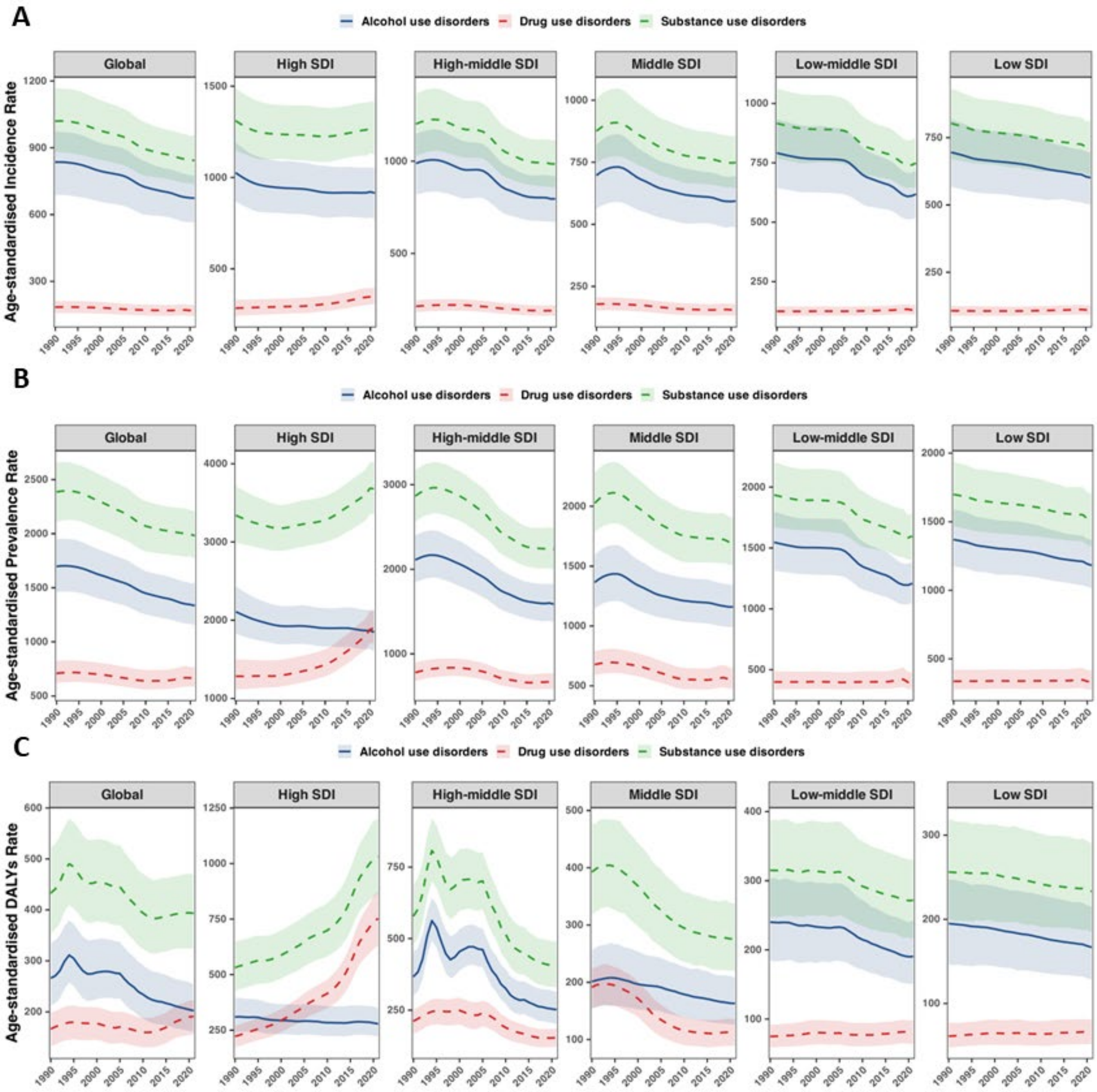

Figure S1. Age-standardised (A) incidence, (B) prevalence, and (C) DALY rates of substance use disorders by Socio-demographic Index, 1990–2021. Abbreviations: SDI=Socio-demographic Index.

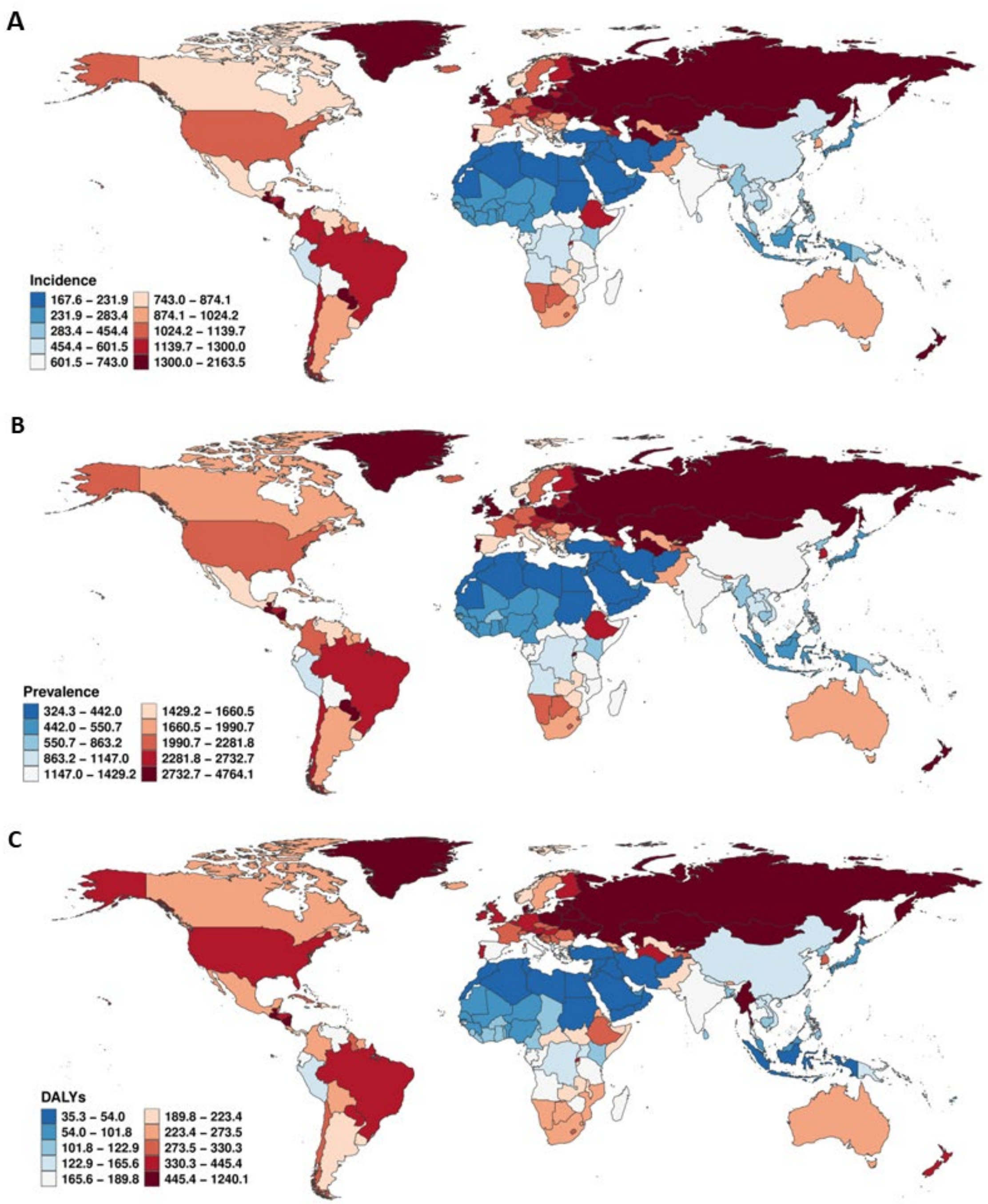

Figure S2. Global age-standardised (A) incidence, (B) prevalence, and (C) DALY rates of alcohol use disorders, 2021. Abbreviations: DALY=disability-adjusted life year.

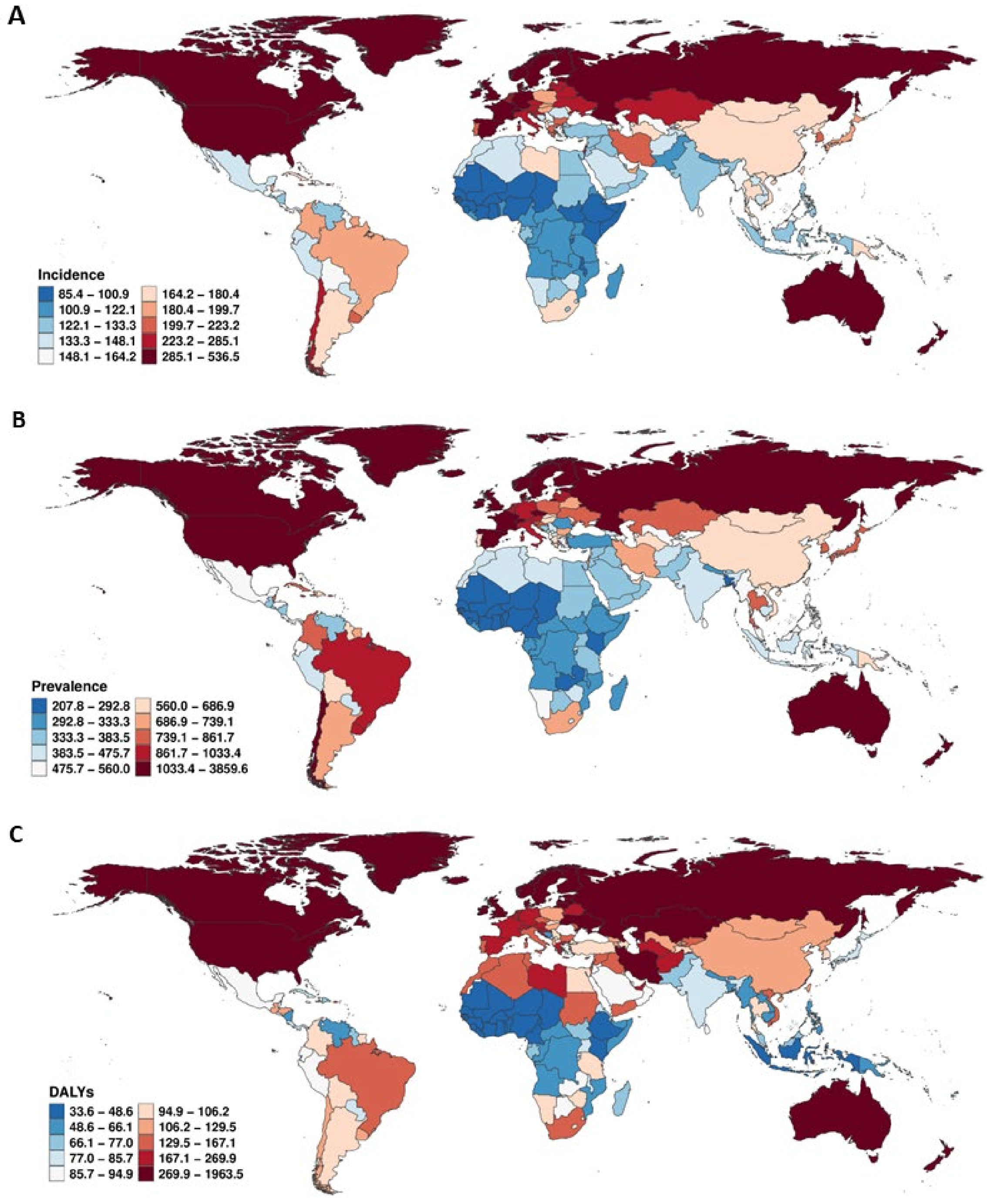

Figure S3. Global age-standardised (A) incidence, (B) prevalence, and (C) DALY rates of drug use disorders, 2021. Abbreviations: DALY=disability-adjusted life year.

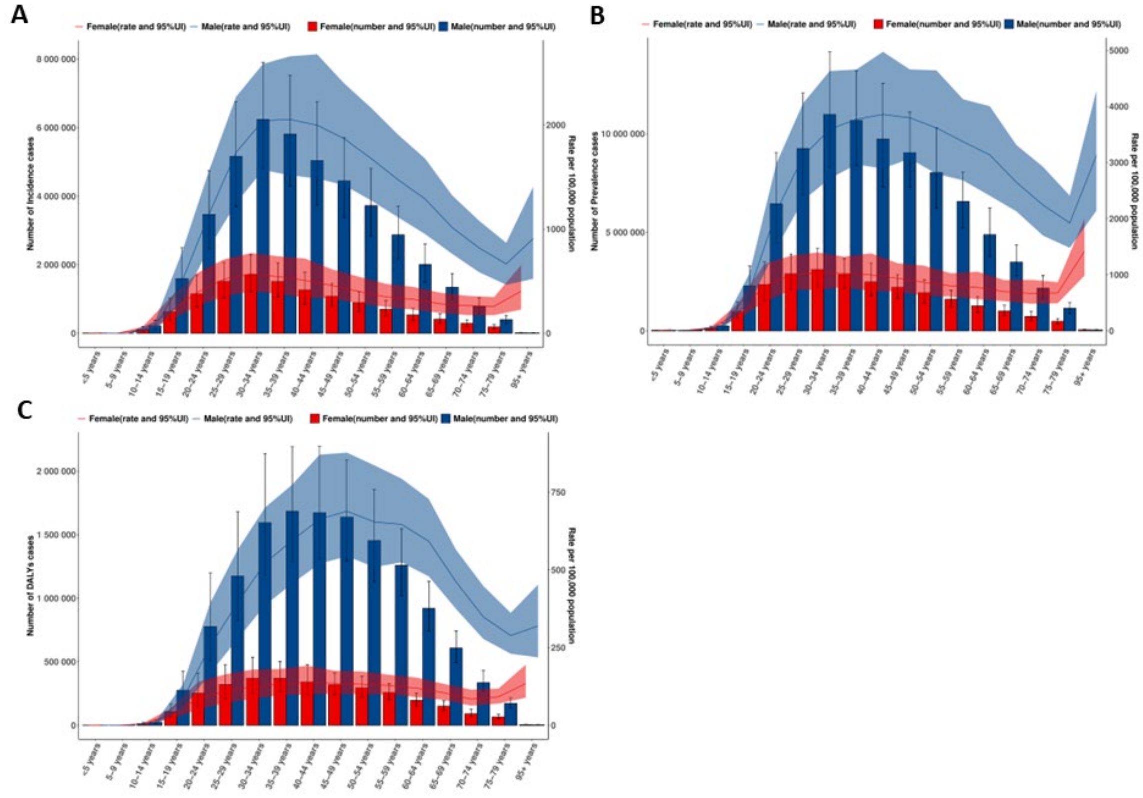

Figure S4. Age-sex patterns of alcohol use disorder burden, 2021. (A) Incidence, (B) Prevalence, (C) DALYs. Abbreviations: DALY=disability-adjusted life year.

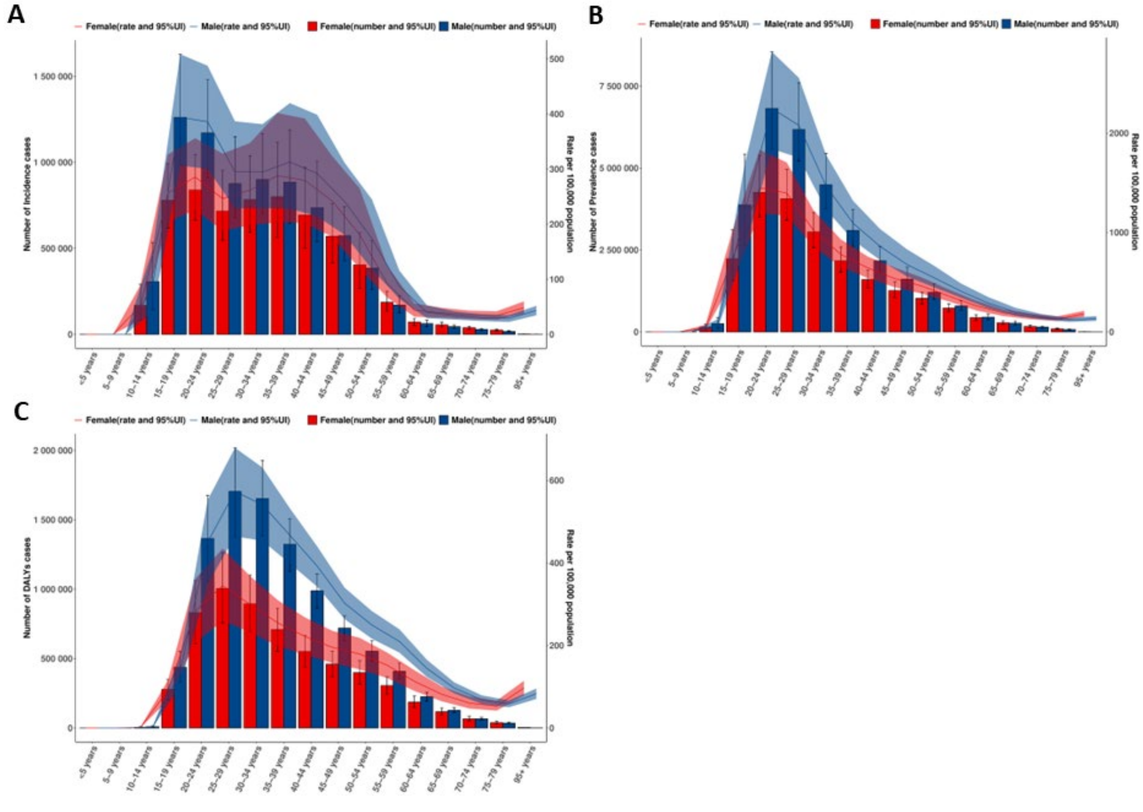

Figure S5. Age-sex patterns of drug use disorder burden, 2021. (A) Incidence, (B) Prevalence, (C) DALYs. Abbreviations: DALY=disability-adjusted life year.

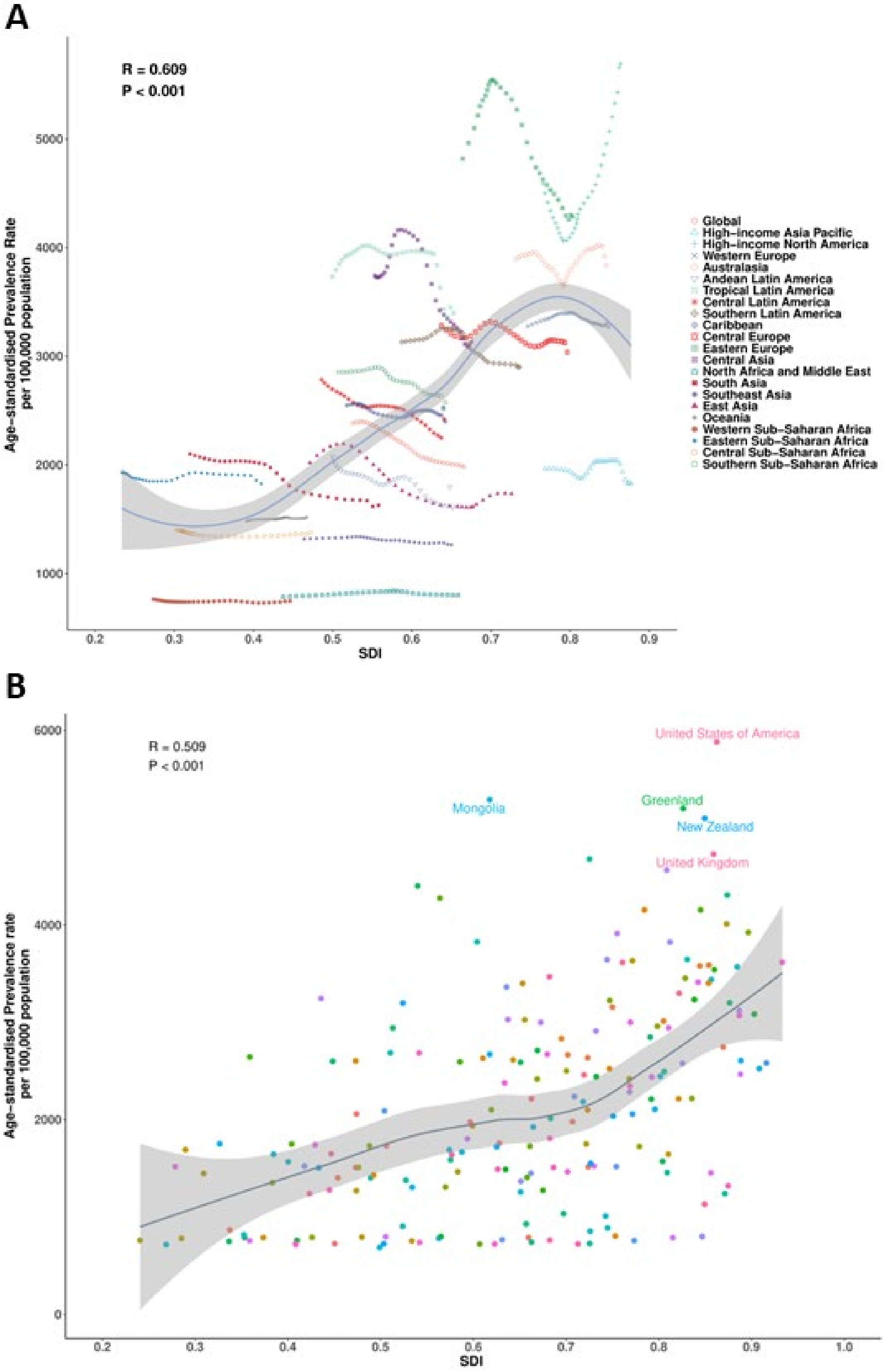

Figure S6. Association between Socio-demographic Index and age-standardised prevalence of substance use disorders, 2021. (A) 21 Global Burden of Disease regions. (B) 204 countries and territories. Abbreviations: SDI=Socio-demographic Index.

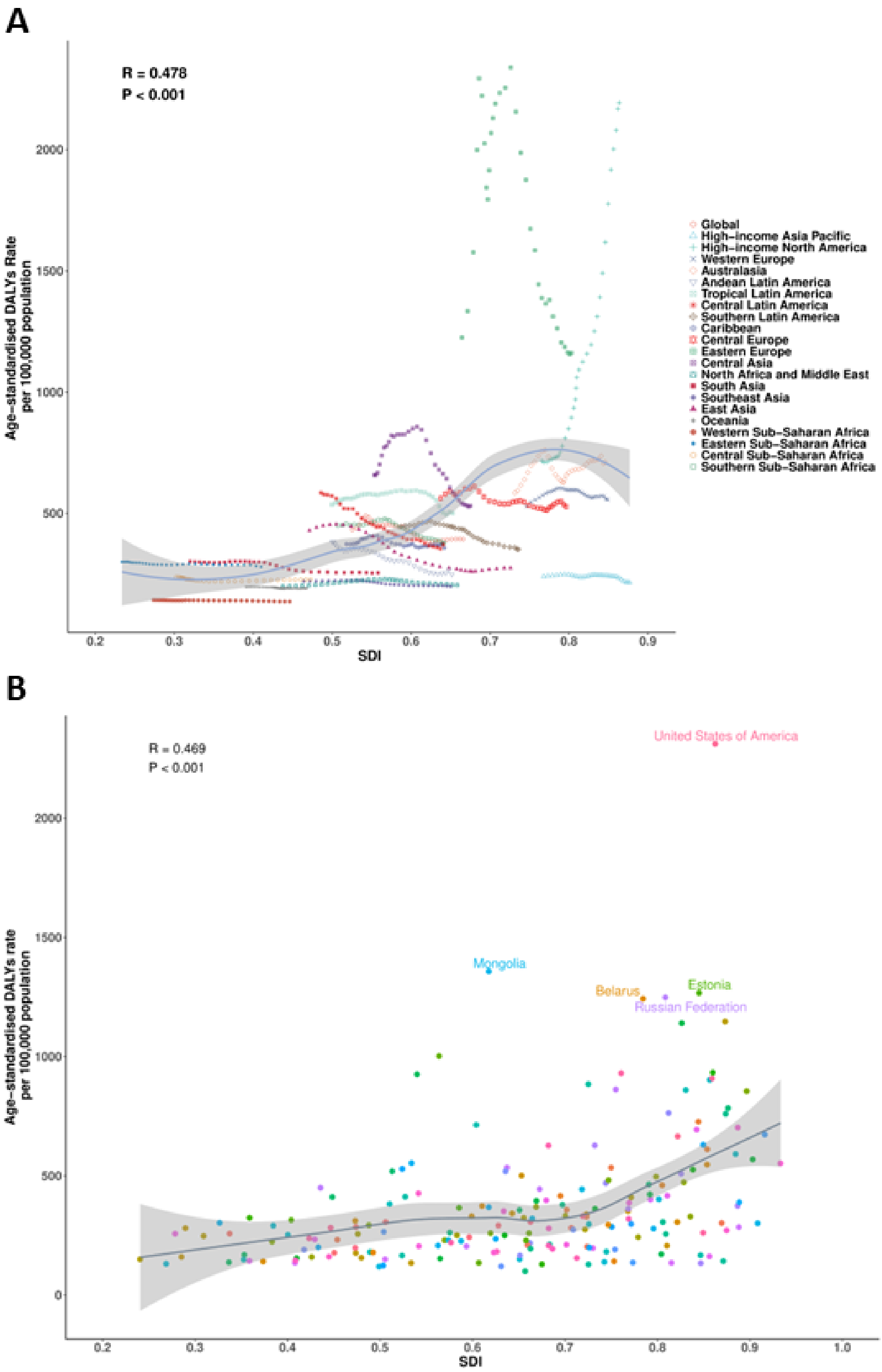

Figure S7. Association between Socio-demographic Index and age-standardised DALY rates of substance use disorders, 2021. (A) 21 Global Burden of Disease regions. (B) 204 countries and territories. Abbreviations: SDI=Socio-demographic Index; DALY=disability-adjusted life year.

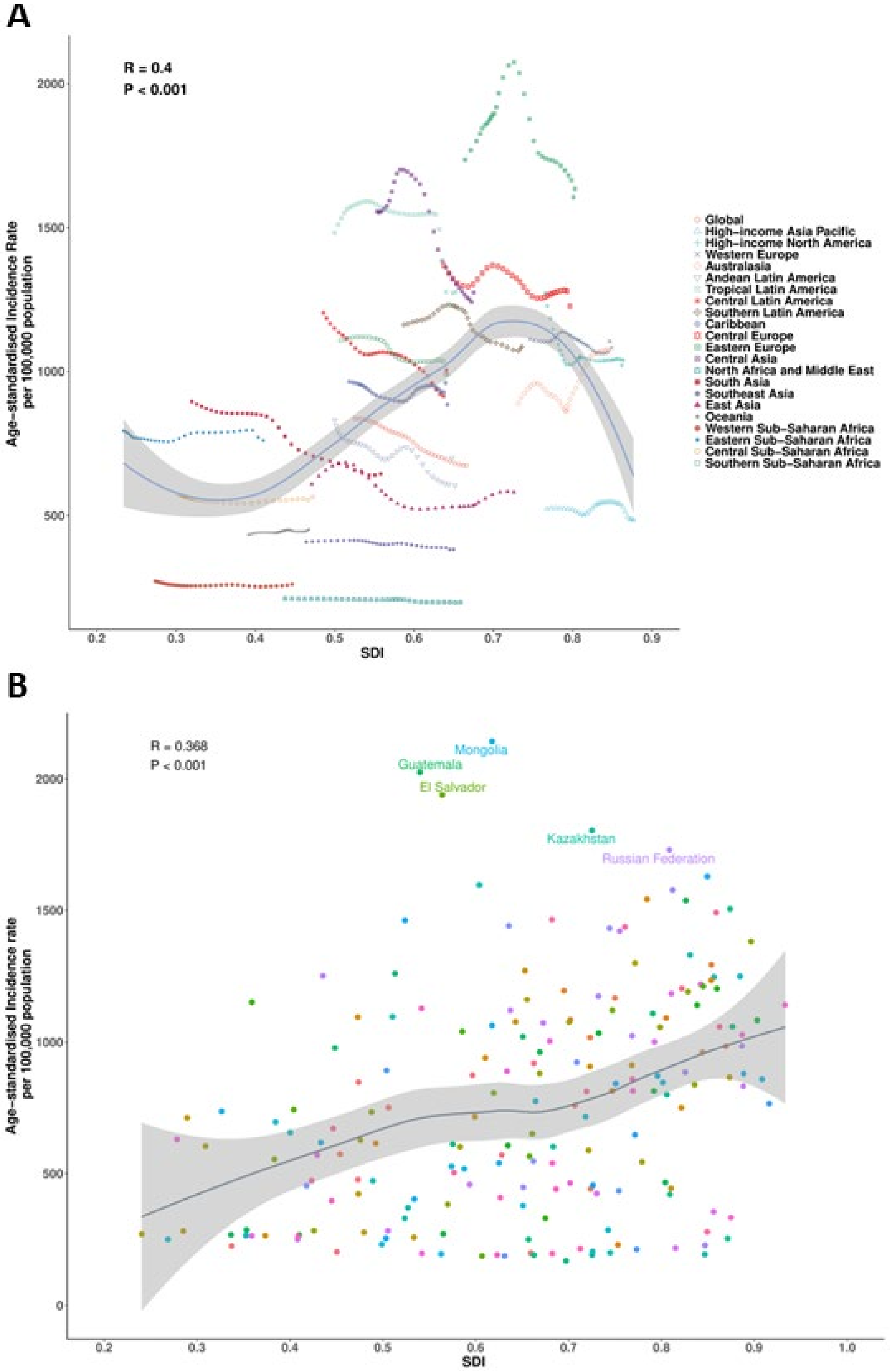

Figure S8. Association between Socio-demographic Index and age-standardised incidence of alcohol use disorders, 2021. (A) 21 Global Burden of Disease regions. (B) 204 countries and territories. Abbreviations: SDI=Socio-demographic Index.

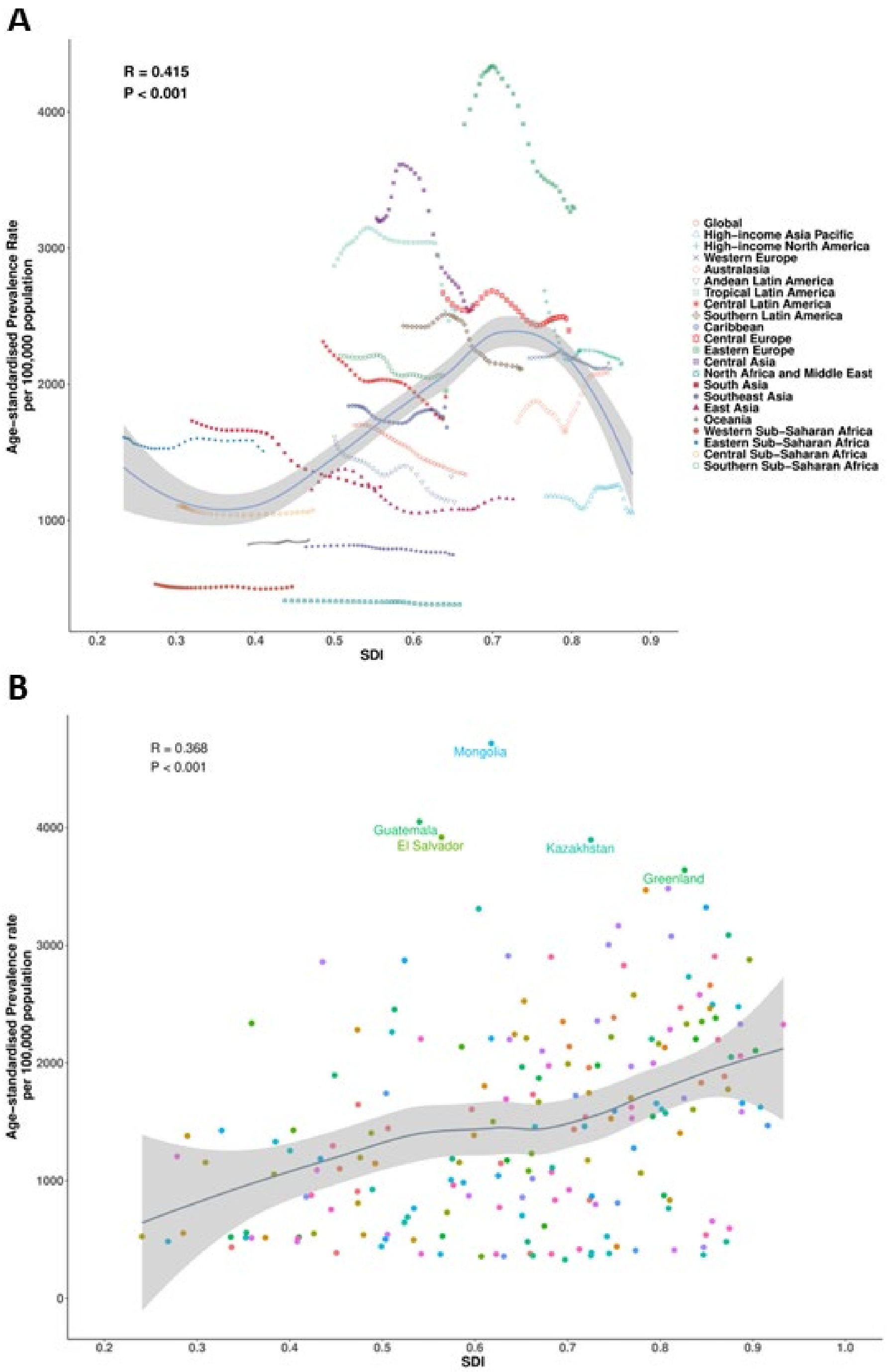

Figure S9. Association between Socio-demographic Index and age-standardised prevalence of alcohol use disorders, 2021. (A) 21 Global Burden of Disease regions. (B) 204 countries and territories. Abbreviations: SDI=Socio-demographic Index.

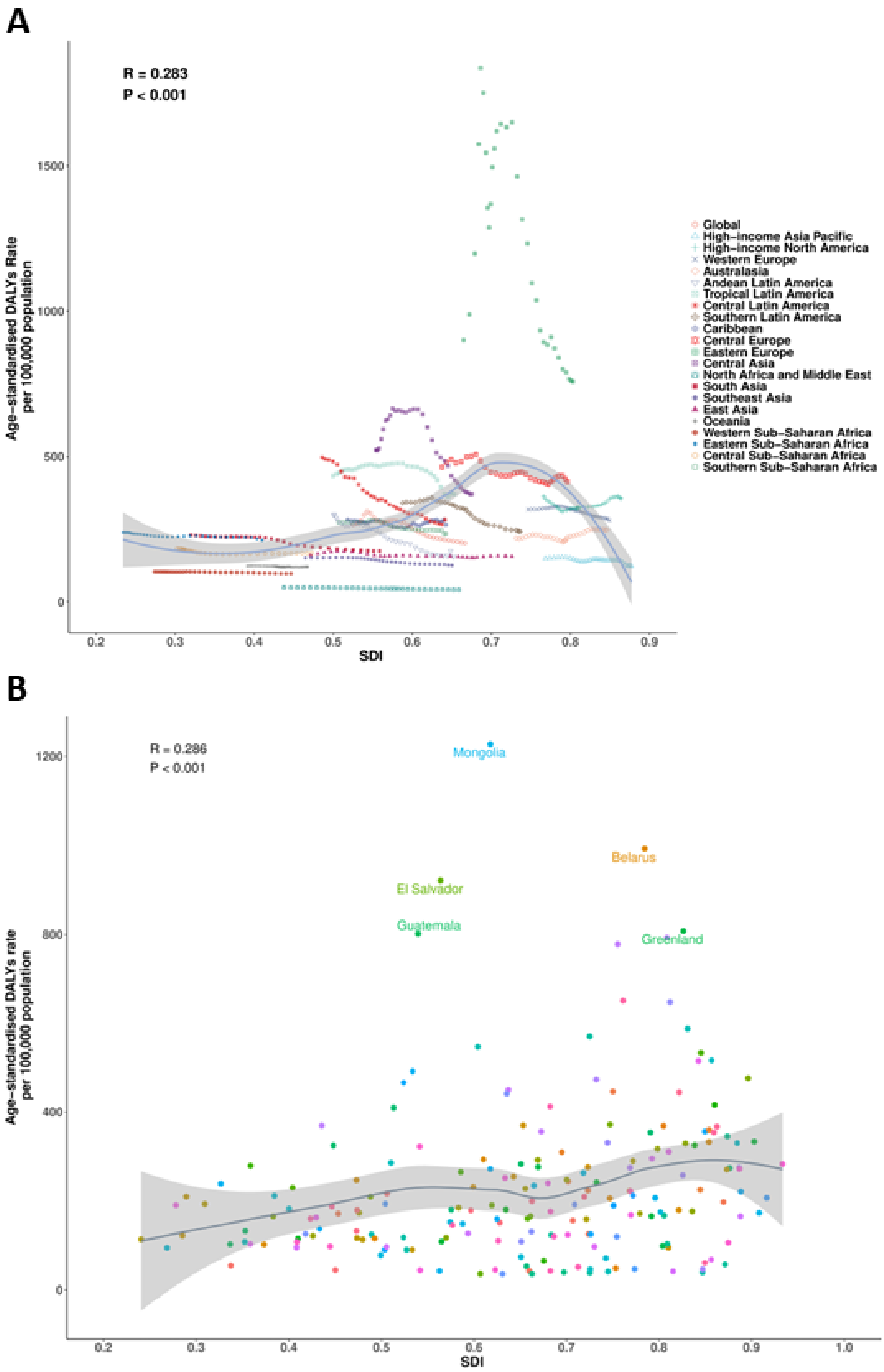

Figure S10. Association between Socio-demographic Index and age-standardised DALY rates of alcohol use disorders, 2021. (A) 21 Global Burden of Disease regions. (B) 204 countries and territories. Abbreviations: SDI=Socio-demographic Index; DALY=disability-adjusted life year.

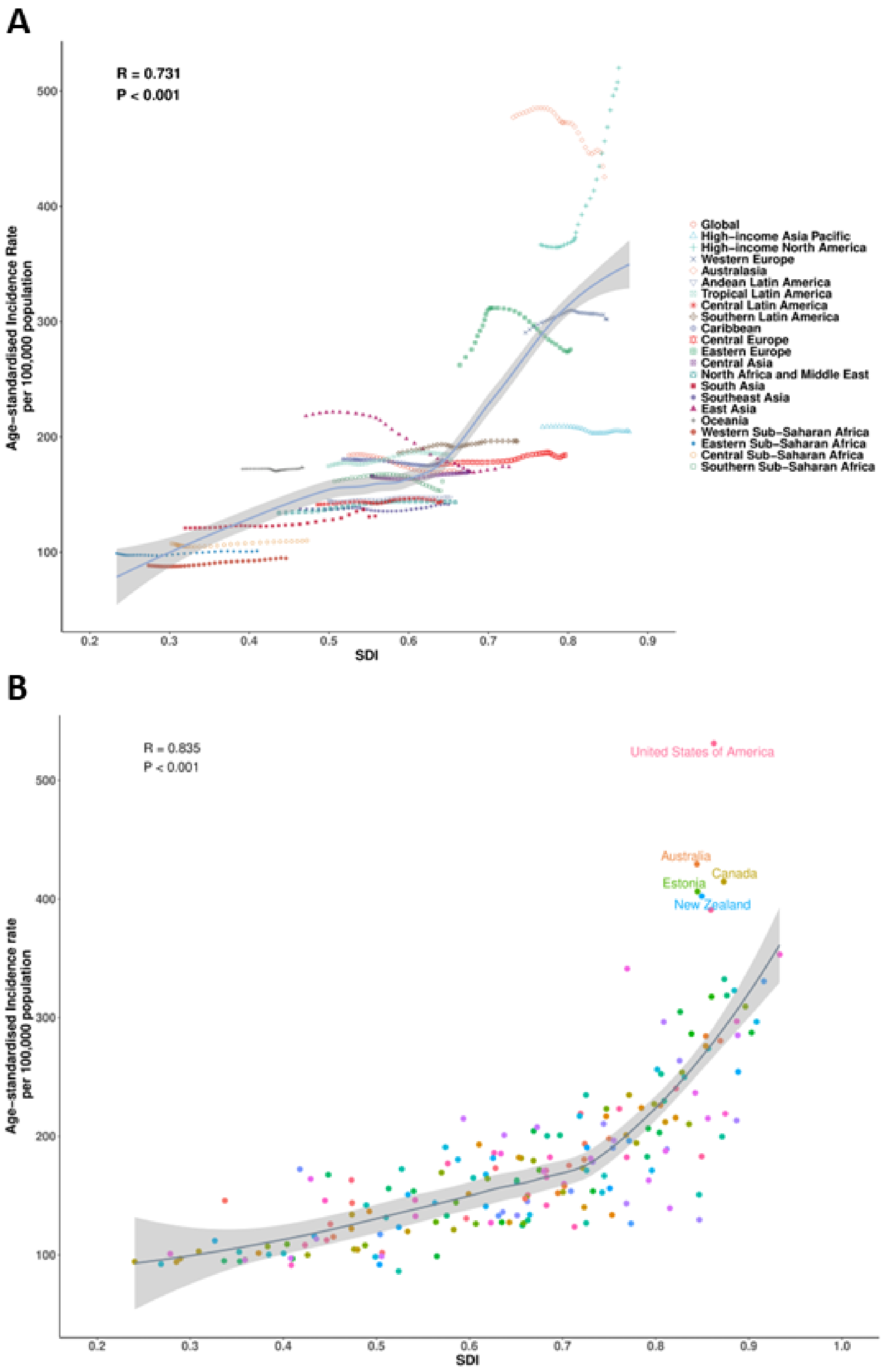

Figure S11. Association between Socio-demographic Index and age-standardised incidence of drug use disorders, 2021. (A) 21 Global Burden of Disease regions. (B) 204 countries and territories. Abbreviations: SDI=Socio-demographic Index.

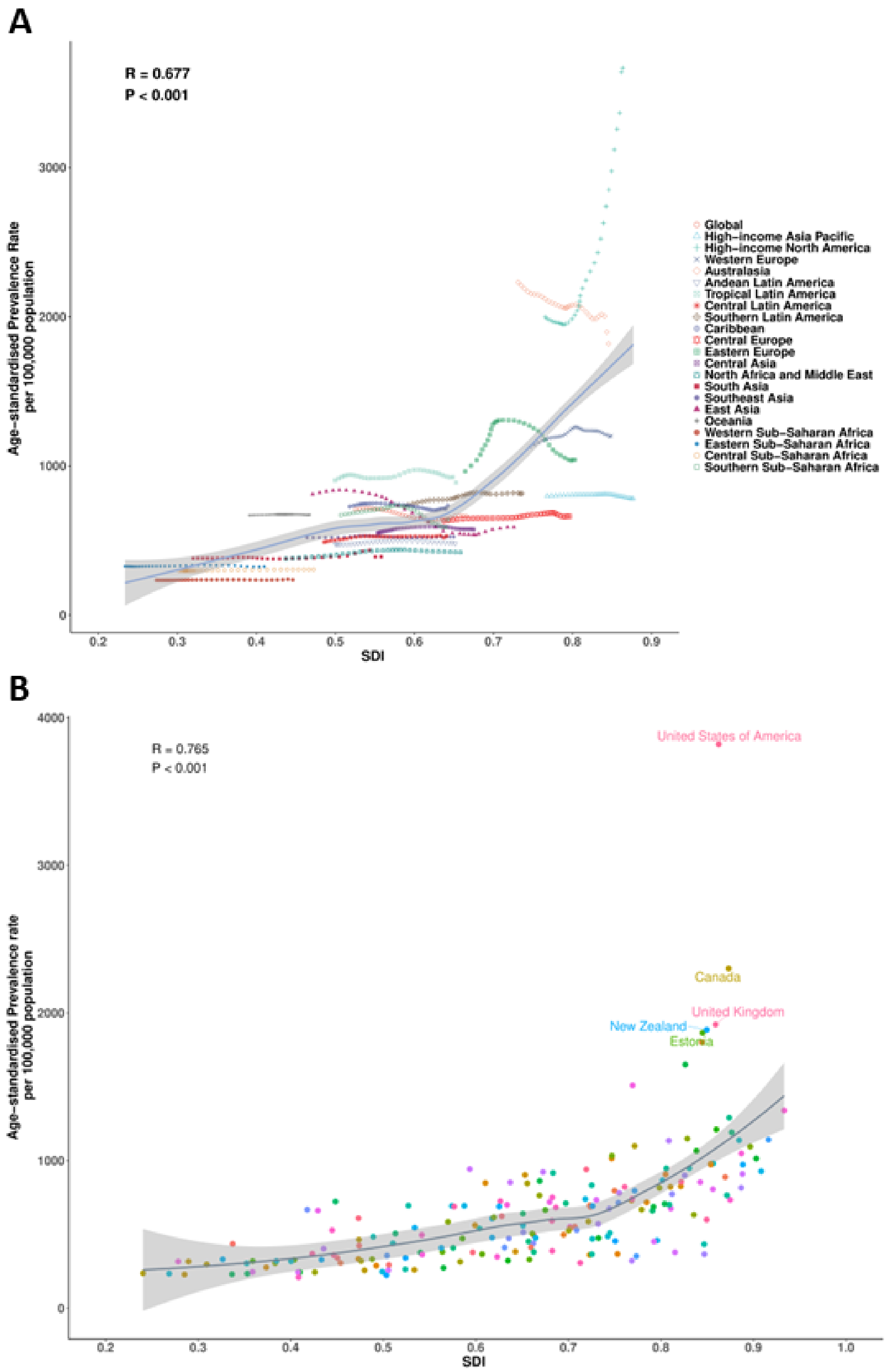

Figure S12. Association between Socio-demographic Index and age-standardised prevalence of drug use disorders, 2021. (A) 21 Global Burden of Disease regions. (B) 204 countries and territories. Abbreviations: SDI=Socio-demographic Index.

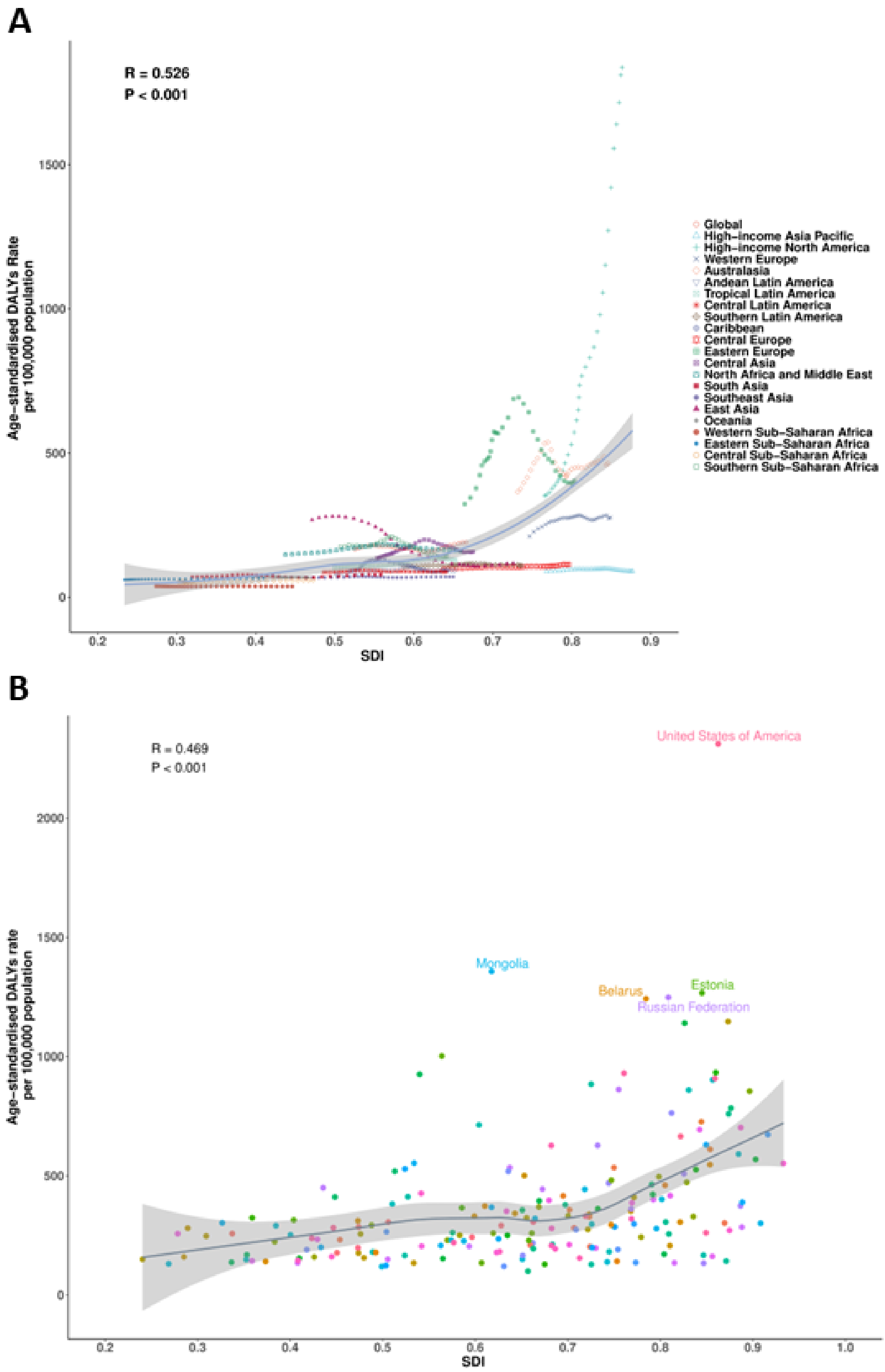

Figure S13. Association between Socio-demographic Index and age-standardised DALY rates of drug use disorders, 2021. (A) 21 Global Burden of Disease regions. (B) 204 countries and territories. Abbreviations: SDI=Socio-demographic Index; DALY=disability-adjusted life year.
